# Risk factors and predictors for Lewy body dementia: a systematic review

**DOI:** 10.1038/s44400-025-00022-2

**Published:** 2025-08-04

**Authors:** Ahalya Ratnavel, Francesca R. Dino, Celina Jiang, Sarah Azmy, Kathryn A. Wyman-Chick, Ece Bayram

**Affiliations:** 1 Department of Molecular, Cellular, and Developmental Biology, University of California Santa Barbara, Santa Barbara, CA USA; 2 Department of Neurology, University of Colorado Anschutz, Aurora, CO USA; 3 Department of Neuroscience, University of Pennsylvania, Philadelphia, PA USA; 4 Department of Neurology, Struthers Parkinson’s Center and Center for Memory and Aging, HealthPartners/Park Nicollet, Bloomington, MN USA

**Keywords:** Neurological disorders, Predictive markers

## Abstract

Lewy body dementia (LBD), including Parkinson’s disease dementia (PDD) and dementia with Lewy bodies (DLB), is a common and burdensome dementia. Determining risk factors and predictors can provide insights into pathogenesis and guide treatment efforts. In this systematic review, we searched PubMed, Embase, and Web of Science for longitudinal studies assessing risk/prodromal factors; including participants without dementia at baseline; with LBD as the outcome; with good/high quality based on the Newcastle-Ottawa Quality Assessment Scale. Across 167 included studies, more consistently reported factors were older age, male sex, *APOEe4*, *GBA*, changes in cognition, mood, behavior, sleep, gait/posture, speech, parkinsonism, smell loss, autonomic dysfunction, white matter disease on MRI, lower CSF amyloid β42 and higher CSF/blood neurofilament light chain. The majority focused on clinical factors preceding PDD with cohorts from North America and Europe, limiting generalizability. Further efforts with more representative cohorts are needed to better identify people at risk for LBD.

## Introduction

Lewy body dementia (LBD), including dementia with Lewy bodies (DLB) and Parkinson’s disease dementia (PDD), is the second most common neurodegenerative dementia^1^. Parkinson’s disease (PD) is the fastest growing neurological disease globally, with up to 80% of the affected individuals at risk for dementia development during disease course^2,3^. Within dementia cases, DLB can account for 7.5% and PDD can account for 3.6%^4,5^. Both dementias include a range of cognitive, behavioral, and motor symptoms with substantial burden on the individuals, their loved ones, healthcare, and the society^1,6^. Clinically, PD and DLB can be differentiated by the interval between dementia and parkinsonism onset. For PDD, dementia occurs at least a year after the onset of parkinsonism^7^. For DLB, dementia can occur before, at the same time, or within the first year of parkinsonism^8^. Both diseases share many overlapping features and are pathologically diagnosed by alpha-synuclein aggregation with Lewy bodies and Lewy neurites in the brain^1^. Accordingly, PDD and DLB are at times grouped together as LBD in research cohorts.

Despite the growing prevalence and significant burden associated with LBD, only symptomatic treatments providing limited relief are available. Disease modification remains the priority in LBD research^9,10^. However, these efforts are limited by the diagnostic challenges at the clinic. Clinical heterogeneity for both PDD and DLB underscore the importance of biomarkers to improve the diagnostic accuracy. Fortunately, recent advances in biomarkers are promising^10^. In fact, these biomarkers detecting the underlying synucleinopathy and neurodegenerative changes can help identify people even before the development of dementia^10^. Identifying people in the prodromal stages can increase the chances of prevention. Furthermore, a better understanding of risk factors can provide more insight into disease mechanisms to help improve both the diagnosis and treatment efforts. The number and strength of predictive models continuously increase in Alzheimer’s disease (AD) and dementia^11,12^. While our aim in this review is not to build and validate a risk/predictive model for LBD, a summary of literature for the risk and predictive factors in LBD can lead to future efforts to develop models for clinic and research. To support such efforts, we conducted a systematic review examining the risk factors for LBD. We included studies in LBD, as well as individually in PDD and DLB to provide a more comprehensive review of the literature. As the pathophysiology of LBD likely involves multiple mechanisms, we report a summary of studies on non-modifiable (e.g., age, sex, genetics), modifiable (e.g., education, health conditions, medications, lifestyle factors) and clinical (e.g., clinical signs, imaging, biomarkers) risk and predictive factors.

## Results

**Fig. 1:**
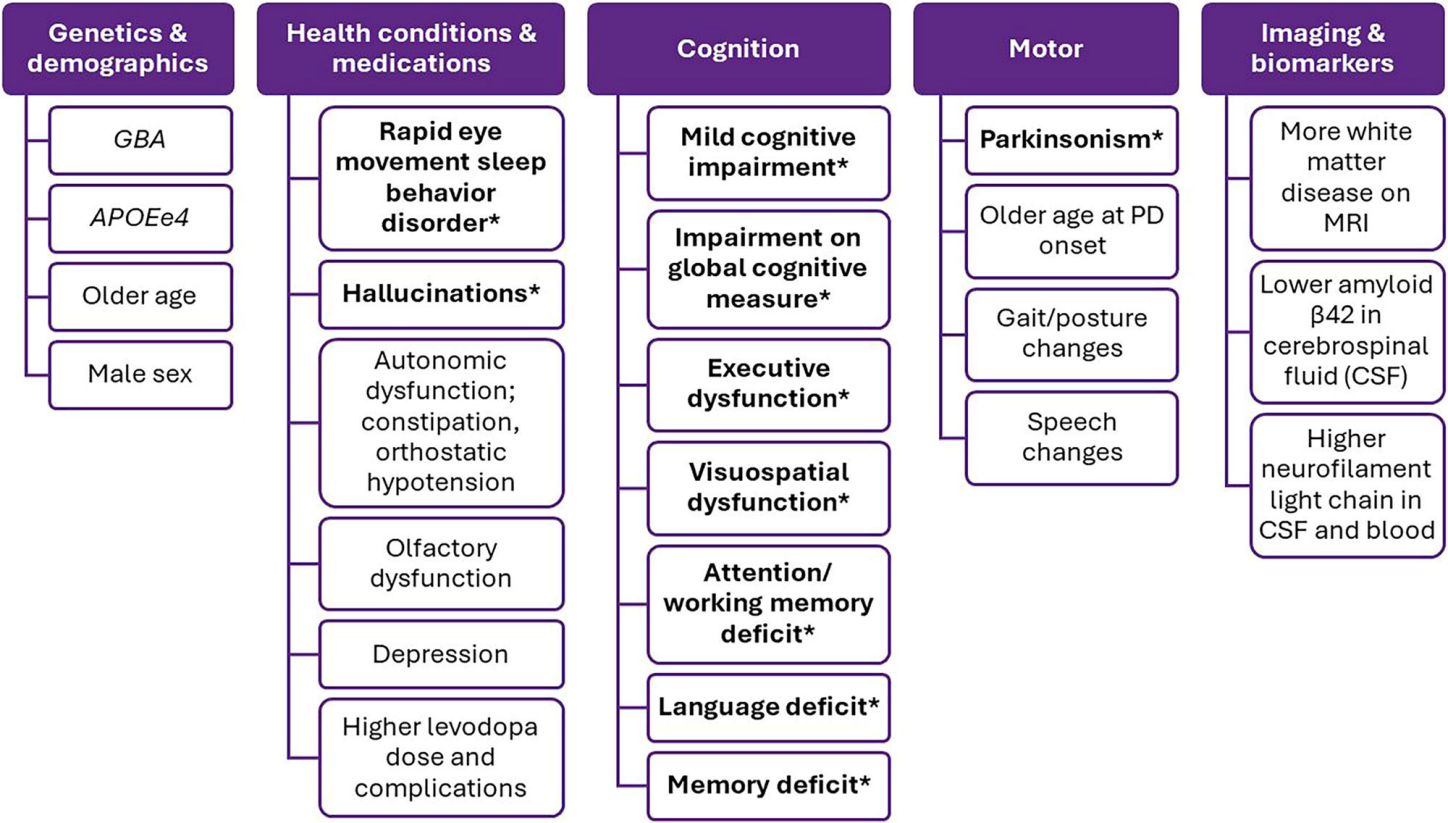
Factors associated with Lewy body dementia reported in at least 2 studies after considering contradicting reports and overlapping cohorts. All factors were reported in Parkinson’s disease dementia (PDD). Factors reported for both PDD and dementia with Lewy bodies are bolded and marked with *.

**Fig. 2:**
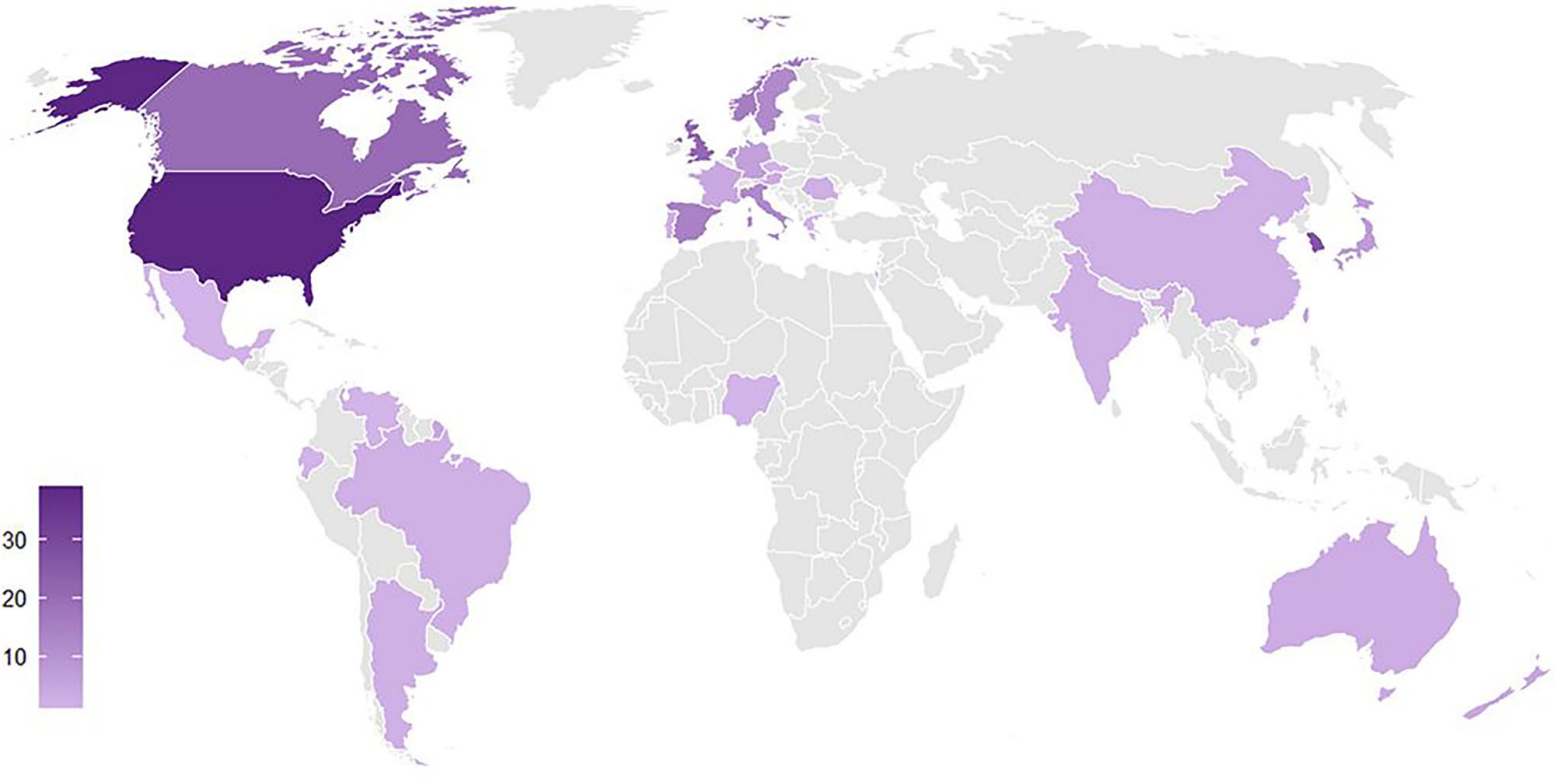
Number of publications from each country.

Longitudinal studies including participants without dementia at baseline with DLB, PDD, or LBD as the outcome assessing a risk, prodromal, or predictive factor were identified through searches of PubMed, Embase, and Web of Science for peer-reviewed articles published by July 19th, 2024. The details of the included 167 studies are summarized in Fig. 1 and Supplementary Tables 1–4. All included studies for data extraction and synthesis had good/high quality based on the Newcastle-Ottawa Quality Assessment Scale. Majority of the studies focused on PDD as the outcome (86.2%, *n* = 144), with a smaller number of studies for DLB (12.0%, *n* = 20) and LBD (1.8%, *n* = 3). The majority were conducted in cohorts from a single country (91.6%, *n* = 153), with a small number including cohorts from different countries (8.4%, *n* = 14). Only one study included participants from Africa; three studies included participants from South America; seven included participants from Oceania; 44 included participants from Asia; 52 included participants from North America; and 79 included participants from Europe (Fig. 2). Sample sizes for participants assessed for risk ranged from 15 to 437,045 people.

Two studies did not report age at baseline; two did not report sex ratios; five did not report the clinical diagnostic criteria for dementia. Mean follow-up duration ranged from 1 to 12 years. Mean age at baseline for the participants at risk ranged from 57 to 81 years. One study was conducted only in males, one study was conducted only in females. Out of the rest of the 163 studies, the ratios for females in the cohorts ranged from 11.4% to 72.6%. Out of the 162 studies, the majority (89.5%, *n* = 145) utilized clinical diagnostic criteria/medical record diagnostic codes, and 10.5% (*n* = 17) utilized global cognitive screening tools for the diagnosis of dementia. Out of the 167 studies, 31.1% investigated non-modifiable (total *n* = 52, 33 demographic, 24 genetic; all with PDD; Supplementary Table 1), 34.7% investigated modifiable (total *n* = 58, 15 medication, 43 health condition, eight social; Supplementary Table 2), and 65.9% investigated clinical factors (total *n* = 110; 77 clinical signs and symptoms, 30 imaging, 19 fluid biomarkers; Supplementary Tables 3, 4) (Fig. 1 for the summary).

### Studies for PDD

For dementia risk in PD cohorts, 52 studies included non-modifiable, 50 included modifiable, and 96 included clinical factors (Fig. 1, Supplementary Tables 1–4). Most used diagnostic criteria (56.3%) to identify PDD in the studies was the MDS PDD criteria^7^. Other diagnostic approaches included Diagnostic and Statistical Manual of Mental Disorders (DSM) criteria, International Classification of Diseases (ICD) codes, and global cognitive screening measures (e.g., Mini Mental State Examination [MMSE], Montreal Cognitive Assessment [MoCA]).

### Non-modifiable factors for PDD

Older age^13–37^, and male sex^14,15,36,38–41^ were the demographic factors associated with PDD risk. There were also studies contradicting these reports; one study reported younger age was associated with increased dementia^42^, and several studies reported no significant associations for sex^17,30,42–45^.

One of the most reported genetic risk factors was *GBA*^40,46–52^. Only one study did not identify a significant association between *GBA* and PDD risk^53^. Severe *GBA1* mutations increased PDD risk more than mild mutations^47^. One study noted a sex interaction; males with *GBA* mutations had a higher PDD risk than females^40^. Another study noted an interaction with *APOEe4*; being a carrier of both *APOEe4* and *GBA* mutations increased risk more than only one risk genotype^51^. While two studies reported *APOE* was not significantly associated with risk^40,54^, more reported *APOEe4* as a risk factor for PDD^44,45,51–53,55^. There was no sex interaction with *APOE* for dementia risk^44^. Three studies with a sample size ~100 people with PD, including two studies with the same cohort, reported *MAPT* H1/H1 genotype as a PDD risk factor^22,35,56^. However, a more recent study with a sample size of 1002 people with PD, including the cohort from those two studies with the same cohort and also other cohorts from UK, Sweden, and Norway, did not find *MAPT* significant^51^. High COMT activity haplotypes were associated with higher risk^57^, while two studies did not find significant associations between *COMT* and PDD risk^35,58^. Other reported risk factors included long leukocyte telomere length^59^, *PITX3* C allele^60^, *SNCA* Rep1 263 allele^61^, *DRD2* ^957^T/T genotype^58^, *RIMS2*, *TMEM108*, and *WWOX*^52^. *AQP4* A allele was associated with slower conversion^62^. *SNCA* (rs356219) was not significantly associated with dementia risk^51^. Shorter *TOMM40* ‘523’ poly-T repeat was associated with higher risk only for female *APOEe3/e3* carriers, with this finding becoming nonsignificant after Bonferroni correction^63^. Lysosomal pathway-specific polygenic risk score also predicted earlier progression to dementia in people with PD with a low likelihood for AD co-pathology^64^.

### Modifiable factors for PDD

Significant risk factors for PDD included lower years of education^28,65^, frailty^66^, smoking^67,68^, constipation^13,69^, insomnia^70^, rapid eye movement sleep behavior disorder (RBD)^13,14,31,37,45,71–73^, orthostatic hypotension^14,15,71,74^, autonomic dysfunction^70,75^, hypertension^76^, dyslipidemia^76^, diabetes mellitus^13^, chronic kidney disease^77^, chronic obstructive pulmonary disease^77^, stroke^77^, bipolar spectrum disorder^78^, delirium^79^, abnormal color vision^71^, abnormal stereopsis^13^, and olfactory dysfunction^13,80–82^. Detrusor overactivity was associated with higher dementia risk in a cohort of only females with PD^83^. High variability in fasting glucose across visits was associated with PDD risk, even among individuals without diabetes^84^. Among people with metabolic syndrome, those treated for hypertension, diabetes, and hypertriglyceridemia had a lower risk for PDD compared to no treatment^85^. Behavioral problems including impulse control disorders^74^, mood changes (i.e., depression^13,33,48,76^, apathy^28^), psychosis (i.e., hallucinations^14,43,86–91^, delusions^89^), and hyperactivity increased risk^92^. Lack of phone use, used as a measure of social engagement, predicted PDD^20^. There were also studies reporting education^30,43^, diabetes mellitus^68,93^, kidney disease or estimated glomerular filtration rate^94^, olfactory dysfunction^71^, depression^30^, smoking, hypertension, stroke/transient ischemic attack, coronary heart disease, and atrial fibrillation^93^ were not significant factors. Compared to being under-/normal-weight, overweight/obesity was associated with a lower risk for PDD^95^.

Chinese herbal medicine^77^, and dihydropyridine calcium channel blocker^96^ were associated with lower risk. Statins were associated with lower risk in one study^77^, and higher risk in another study^97^. Although anticholinergics were not individually associated with dementia risk^98^, cumulative dose effect^98^ and exposure for 6 months or more^99^ increased PDD risk. These two studies for anticholinergics were conducted using data from the National Health Insurance Research Database of Taiwan, however the cohorts were not fully overlapping^98,99^. Higher levodopa dose^18,28^, levodopa induced dyskinesia^100^, confusion or psychosis^33^ were also associated with higher PDD risk.

### Clinical factors for PDD

Subjective cognitive decline^45^, difficulty performing instrumental activities of daily living^101^ and mild cognitive impairment (MCI)^14,15,21,23,27,36,71,81,102–109^ predicted PDD. Multi-domain MCI was associated with higher risk than single domain^107,109,110^ with executive and attention deficits as the stronger predictors^107^. For people with single domain MCI, one report showed a higher dementia risk for non-amnestic than amnestic profile^111^, while another report reported the opposite with higher dementia risk for amnestic than non-amnestic profile^112^. Impairment on global cognitive measures^29,67^ including MMSE^25,45,48,74,102,113^, MoCA^102^, and clock drawing^114^ predicted PDD. Neuropsychological test performances indicating worse function for executive^30,32,109,110,115–120^, visuospatial^27,35,80,109,110,115,118,119,121,122^, attention/working memory^34,109,110,119,122–124^, processing speed^34,114,123^, memory^32,34,43,109,115,119,125,126^ and language^43,109,115,119,122,124,126^ were also found to predict PDD.

Older age at PD onset^39,40,43,48,65,76,118^, more severe motor symptoms^14,16–19,21,28,30,33,34,36,37,39,43,45,48,67,81,113,125,127,128^ and non-tremor-dominant subtype^37,86,87,91,129,130^ were associated with higher PDD risk. Individual motor symptoms associated with risk included changes in gait/posture^14,34,71,87,131^, speech^67,87,132^, rigidity^87^, upper limb bradykinesia^67^, masked face^33^. There were a lower number of studies reporting age at PD onset^16,26,27,128^, motor symptom severity^27^, and PD subtype were not significant factors^121^. More frequent and severe non-motor symptoms posing more burden on the individual than motor symptoms also increased the PDD risk^133^.

Frontal and anterior cingulate cortical thinning^122^, more white matter disease burden^41,108,131,134^, larger choroid plexus volume^135^, larger third ventricle volume^34^, reduced hippocampal volume^136^, reduced nucleus basalis of Meynert volume^137^, and more heterogeneity in the caudate texture on the less-affected side^32^ were identified as PDD predictors in magnetic resonance imaging (MRI) studies. Higher scores on the grand total of electroencephalogram (EEG) scale, with more diffuse slowness and focal disturbances were associated with PDD risk^138^. Rapid eye movement sleep and wakefulness slowing ratios in temporal and occipital regions, and dominant occipital frequency predicted PDD^139^. Low cardiac iodine-123-meta-iodobenzylguanidine (MIBG) uptake (H/M ratio < 1.35)^24^; low beta power on magnetoencephalogram (MEG) combined with impairment on executive tests were significant predictors^116^. Atypical fludeoxyglucose positron emission tomography (FDG PET) pattern, such as AD-like pattern or DLB-like pattern, characterized by metabolic alterations in the posterior parietal-occipital regions, were associated with higher risk in a PD cohort from Italy with dementia outcome within four- and eight-year time points^140,141^. Two ^18^F-FP-CIT PET studies with overlapping cohorts from South Korea noted reduced uptake in the anterior putamen as a PDD predictor^41,142^, while one study found no significant association for putaminal dopamine uptake^143^. Other significant predictors with ^18^F-FP-CIT PET included low cingulate island sign ratio (lower regional uptake in posterior cingulate relative to the precuneus and cuneus compared to mean ratio for controls)^144^, lower uptake in posterior putamen^127^, higher cerebral perfusion on early phase scans^145^, and reduced uptake in frontal, parietal, temporal, and lateral occipital regions compared to preserved cortical uptake^146^. For people with MCI, who subsequently developed PD with half of them converting to PDD during the ~5-year follow-up period, PDD risk was associated with reduced putamen-to-caudate striatal binding ratio in the less affected hemisphere with dopamine transporter single photon emission computed tomography (SPECT), coupled with hypometabolism of temporal lobes on FDG PET^124^.

The most reported cerebrospinal fluid (CSF) biomarker for PDD was lower levels of amyloid β^23,55,81,122,133,147,148^. Specifically, amyloid β42 was noted as a risk factor^23,55,81,148^, while amyloid β40 and amyloid β38 were not associated with risk^23^. Higher neurofilament light chain (NfL)^147,149^, higher heart fatty acid-binding protein^147^, higher glial fibrillary acidic protein^150^, and lower glucocerebrosidase activity^151^ were also associated with risk. Total and phosphorylated tau_181_ (p-tau_181_) levels were not individually associated with risk^23,148^, although p-tau_181_ can be helpful for prediction when combined with lower levels of amyloid β42 and higher serum NfL^148^. In addition, ratios of CSF biomarkers, such as higher total tau/alpha synuclein and total tau/amyloid β1-42+alpha synuclein, were significant predictors for PDD^152^. For blood, higher NfL^53,148,153^, lower B12^154^, lower zinc (Zn) levels^155^, higher levels of a panel including hydroxy-isoleucine, His-Asn-Asp-Ser, Alanyl-alanine, Putrescine [-2H], 3,4-Dihydroxyphenylacetone^34^ in the serum, and lower epidermal growth factor levels in the plasma^156^ were associated with higher PDD risk.

### Studies for DLB

For DLB, seven studies investigated modifiable, and 13 investigated clinical factors (Fig. 1, Supplementary Tables 2–4). We did not identify any studies for non-modifiable factors. The majority (75%) were conducted with RBD cohorts. The most used diagnostic approach for DLB was the available McKeith DLB clinical diagnostic criteria at the time of the study (70%)^8,157^. Other diagnostic approaches included DSM criteria and ICD codes.

### Non-modifiable factors for DLB

Herpes simplex virus, but not varicella zoster virus^158^, and adult attention deficit-hyperactivity disorder^159^ were reported as risk factors for DLB. In a US cohort of males, Hart and colleagues focused on medications used for benign prostatic hyperplasia comparing α-1 adrenergic receptor antagonists that also bind to and activate an adenosine triphosphate (ATP)-producing enzyme in glycolysis (terazosin, doxazosin, and alfuzosin) and other medications that do not increase ATP (α-1 adrenergic receptor antagonist tamsulosin, and 5α-reductase inhibitor)^160^. DLB risk was lower for males on terazosin, doxazosin, or alfuzosin compared to males taking tamsulosin or 5α-reductase inhibitor, with similar risk for tamsulosin and 5α-reductase inhibitor. Two studies noted RBD as a risk factor for DLB^161,162^. For people with RBD, cardiovascular disease, hypertension, hypercholesterolemia, and diabetes were not associated with DLB risk^163^. People with RBD with residual injurious symptoms after being treated with clonazepam and/or melatonin had higher DLB risk^164^.

### Clinical factors for DLB

Conversion to dementia for people with RBD was best predicted by attention, executive, and memory tests including Stroop Color Word Test, Trail Making Test Part B, Color Trails Test, digit span backward, verbal fluency, and word learning tests^165–167^. Visuospatial deficits with lower scores on Figure copy^166,167^, false noise errors on the noise pareidolia test^168^, overall MCI^166^ and lower MoCA scores^167^ were also predictors for DLB in people with RBD.

For people with MCI, parkinsonism, cognitive fluctuations, RBD, visuospatial deficit, and impaired letter fluency were associated with DLB risk^169^. In a retrospective study including people with DLB and controls, the presence of two and more core clinical features (parkinsonism, cognitive fluctuations, RBD, visual hallucinations); or one or more core clinical features combined with apathy, depression, or anxiety differentiated people with prodromal DLB from controls^170^.

All the studies investigating imaging and biomarkers for risk included RBD cohorts. Increased hippocampal perfusion on SPECT^171^, hyperechogenicity of the substantia nigra on transcranial sonography^172^, and severe phasic electromyography (EMG) activity compared to mild phasic EMG activity^173^ were reported as DLB predictors. Interhemispheric laterality for striatal dopamine transporter binding on dopamine transporter (DaT) SPECT did not predict DLB^174^. Rahayel and colleagues computed a brain-clinical signature combining brain deformation score on MRI and clinical variables^175^. The combination of MCI, and akinetic-rigid motor phenotype as the clinical variables with atrophy in the basal ganglia, thalamus, amygdala, frontotemporal gray and white matter, and subarachnoid/ventricular expansion as the deformation variables predicted DLB.

Lower N-acetylneuraminic acid in glycoproteins in the serum^176^ and lower CSF amyloid β42 levels^177^ predicted DLB. CSF p-tau, CSF total tau and CSF/serum albumin ratio were not significantly associated with risk^177^.

### Studies for LBD

Two studies focused on modifiable, and one study focused on clinical markers for LBD (Supplementary Tables 2, 4). We did not identify any studies focusing on non-modifiable factors. Diagnostic approaches included McKeith 2017 DLB clinical diagnostic criteria^8^, DSM V criteria, ICD 9 Clinical Modification and Read codes.

Both studies for modifiable factors were conducted retrospectively leveraging large national healthcare databases. Prescription of nonsteroidal anti-inflammatory drugs (NSAIDs) and glucocorticoids over 10 years prior to the dementia diagnosis were associated with higher risk for LBD^178^. Cardiovascular diseases treated with anti-hypertensives, cholesterol-lowering agents, and anti-diabetics were associated with lower LBD risk^179^.

In the only prospective study for LBD with an RBD cohort, reduced perfusion flow in precuneus, posterior cingulate, and parietal association cortex on the brain perfusion ^99m^Tc-ECD SPECT was associated with higher risk, without a significant association for the cingulate island sign^180^.

## Discussion

In this systematic review, we identified studies on a range of non-modifiable, modifiable, and clinical risk factors and predictors for LBD, with the majority including cohorts in North America and Europe and focusing on clinical factors and PDD.

Older age and male sex were risk factors for PDD across several studies, with a lower number of studies suggesting otherwise for age and lack of significance for sex. While both PDD and DLB prevalence typically increases with age, with age at onset in the late fifties to early seventies^1^, we did not identify any longitudinal studies focused on demographics for DLB or LBD. Interestingly, Fink and colleagues noted that the sex difference in their analysis for PDD risk disappeared after accounting for sex-specific survival patterns, and modifiable risk factors such as cardiovascular diseases impact PDD risk differently by sex^39^. Similar to PDD, DLB prevalence is also suggested to be higher for males than females, although this sex difference is not consistent across studies and may disappear with older age^181^. Impact of genetic variants on PDD risk can also differ by sex as noted for *GBA*^40^, but not *APOE*^44^. Thus, the interplay between biological factors needs to be considered for LBD risk.

More frequently studied genetic factors were *GBA*, *APOE*, and *MAPT*. There is an increasing number of studies focused on genetic risk in LBD; however, these studies were mostly cross-sectional and thus not included in our review. The most recent work from the International LBD Genomics Consortium showed *GBA*, *BIN1*, *TMEM175*, *SNCA-AS1*, and *APOE* were associated with LBD risk^182^. However, this cohort consisted of participants with European ancestry, and these genes were not found to be significant in a smaller study consisting of participants from Japan^183^. This study by Kimura and colleagues identified another gene, *CDH23*, to be associated with LBD risk^183^. Different findings in these two recent studies underscore the importance of investigating LBD genetic risk factors in different populations. As having both *APOEe4* and *GBA* mutations further enhanced PDD risk^51^, utilization of polygenic risk scores to sum the effects of multiple variants can be helpful, although they can fall short if not taking gene-gene interactions into account^184^.

Education is frequently associated with better cognitive reserve and lower dementia risk^185^. It can impact dementia risk differently based on gender, ethnic, and racial group^186^. The number of studies focusing on education for PDD risk were limited in our review. Additionally, these studies examined years of education rather than education quality, which can also play a role in dementia risk^187^ and should be investigated for LBD. Lifestyle factors can predict or increase the risk for LBD. Smoking is associated with reduced PD risk^188^, and higher dementia risk^185^. While there are not many studies focused on smoking in LBD, findings suggest a potential association between smoking and higher PDD risk^67,68^. Social isolation is a risk factor for dementia^185^, and we identified one study which indicates lack of phone use can signal progression to dementia in people with PD^20^.

Olfactory dysfunction and RBD are considered predictors for both PDD and DLB^1^, as supported by the studies in our review. The risk level for people with RBD can be even higher if they experience autonomic disturbances such as constipation^13^, or sustain injuries during dream enactment despite symptomatic treatment^164^. Interestingly, cardiovascular diseases were not consistently associated with higher PDD or DLB risk in the studies included in our review, despite being a common risk factor for dementia^185^. Medications may have impacted these findings, as several studies identified a lower LBD risk for people treated for these conditions^77,85,179^. As a range of autonomic disturbances were reported as risk factors for PDD, determining the potential impact of treatments for these changes can provide effective prevention strategies for LBD^75^. Psychiatric onset is included as one of the potential clinical profiles in the research diagnostic criteria for prodromal DLB^189^. It is characterized by affective symptoms followed by psychosis prior to DLB onset^190^. Accordingly, we observed a range of psychiatric symptoms, including depression and hallucination, associated with higher PDD and DLB risk. These conditions occurring during the prodromal PD phase^191^ can also occur after PD onset as the non-motor symptoms of PD and predict PDD. Studies in large cohorts showing herpes simplex virus as a risk factor in DLB^158^ with varicella zoster vaccination as a protective factor for dementia^192^; terazosin, doxazosin, and alfuzosin for benign prostatic hyperplasia^160^, NSAIDs, glucocorticoids^178^, anti-hypertensives, anti-diabetics, and cholesterol-lowering agents^179^ as protective factors can be particularly helpful for disease modification trials in LBD.

Antiparkinsonian medications were also found to be associated with PDD risk. However, the higher total medication dose^98^, longer exposure^99^, and treatment complications^33^ were associated with higher risk, instead of individual medications^98^. Considering that more advanced disease is also associated with higher risk, it is likely that disease severity leading to higher medication dose and treatment complications leads to this medication and dementia connection. We noted that various motor symptoms of PD were reported to predict dementia; tremor-dominant subtype was associated with a lower likelihood for dementia development. However, clinical subtypes are unstable and can change over time^193^. Age at onset for PD was amongst the most reported risk factors, although this association may be due to the general effect of age rather than age of disease onset^26^. Aging related changes coupled with PD pathogenesis increase dementia risk further^194^. Risk factors for dementia may differ for people with younger onset compared to older onset.

Subjective cognitive decline and MCI are prodromal phases for dementia and can provide opportunities for intervention to prevent LBD onset. While executive, attention and visuospatial deficits are more typically associated with LBD, people with prodromal LBD can have a wide range of cognitive domains affected^195^. MCI can be difficult to identify in the absence of objective cognitive testing^196^. Comprehensive neuropsychological assessments can help identify people at risk for LBD. Global cognitive screening tools can also be useful in settings where comprehensive testing in not possible. In addition to cognitive testing, assessing color vision^71^, stereopsis^13^, and noise pareidolia test^168^ may also provide useful predictive information.

Different imaging modalities including MRI, EEG, PET, SPECT, MIBG, MEG, transcranial sonography and EMG were assessed for LBD prediction. The most consistently reported finding for biomarkers for LBD prediction was lower CSF amyloid β42 levels in people with RBD and PD, underscoring the prevalence and impact of AD co-pathology in LBD^197^. However, CSF amyloid β42 cut-off values to detect dementia for AD and PD may differ^198^. Recent reports on biomarkers including seed amplification and real-time quaking-induced conversion (RT-QuIC) assays for alpha-synuclein also have promising findings for LBD^199^. Clinical profile in the prodromal phase of LBD is heterogeneous and overlaps with prodromal phases of AD and other dementias^200^. Accordingly, utilizing biomarkers can increase diagnostic accuracy^1,201^ and better define the risk and predictive factors for LBD in future longitudinal studies. While we reported factors individually, several studies built statistical models combining different factors (see Supplementary Tables 1–4). Access to imaging and biomarker studies differ across settings, however, combining available biomarkers with clinical findings appears to be a helpful approach for prediction.

Our review showed that the current literature primarily consists of studies focused on predictors and PDD with many populations remaining underrepresented. The majority of the studies included in our review were conducted at single sites and included cohorts from North America or Europe. In their meta-analysis for dementia risk in PD, Gibson and colleagues noted that PDD incidence rate was lowest in Asia and highest in North America with significant heterogeneity and without any studies from Africa or South America^202^. The meta-analysis by Hogan and colleagues in 2016 noted that DLB accounts for about 5% of all dementia cases with incidence rates ranging from 0.5 to 1.6 per 1000 person-years^203^. As the research diagnostic criteria for the MCI with Lewy bodies was published in 2020 and are relatively new^189,204^, it is likely that the studies focusing on the prodromal stage of DLB will increase in the upcoming years to better understand the incidence of prodromal DLB in MCI cohorts. For studies included in our review, there were no comparisons for risk across different countries or people from different ethnic and racial groups. This further underscores the need for more diversity in research cohorts and multi-site collaborations for better representation and understanding of the underlying reasons behind different incidence rates. We noted that the PDD and DLB clinical diagnostic criteria were the most used diagnostic approaches. Additionally, 26.3% (*n* = 44) primarily relied on medical record diagnostic codes and 10.2% (*n* = 17) only used global cognitive measures (e.g., MMSE, MoCA) with different cut-offs across the studies for diagnosis. Medical record codes have limited diagnostic accuracy for dementia, AD and vascular dementia^205,206^. The differences across studies for the diagnostic approach can impact the outcomes. In addition, the wide range for sample size, follow-up duration, age at baseline and sex ratio for cohorts limit the generalizability of findings.

In conclusion, research so far suggest a role for genetics, aging, sex, education, infections, health conditions and medications in the increased risk of developing LBD, although replication of many findings is still needed to determine their applicability. Predictive models combining a range of factors, and biomarkers when available, can help identify people at risk for LBD, ultimately benefitting disease modification efforts. However, identifying causal factors and the interactions between risk factors is required to better understand the pathophysiology and guide clinical trial strategies. With promising advances in the diagnosis and treatment approaches for neurodegenerative diseases, more research should focus on risk factors for LBD, particularly DLB. Longitudinal study design, consistency for diagnostic criteria and assessments, and collaborative multi-site cohorts from diverse populations can provide better insight to support all at risk for and living with LBD.

## Methods

This systematic review was conducted according to the Preferred Reporting Items for Systematic Reviews and Meta-Analyses (PRISMA) guidelines^207^. The review protocol was not pre-registered.

### Search strategy

Studies were identified by searches of PubMed, Embase, and Web of Science from inception to July 19th, 2024. The search was limited to original peer-reviewed research articles written in English and including humans rather than animals. Case reports, reviews, meta-analyses, conference abstracts, editorial, opinion papers, book chapters, and preprints were excluded. Search terms were based on the concepts of outcome (dementia, cognitive decline, cognitive impairment) AND disease (Lewy, Parkinson, synuclein) AND risk factors (risk, prediction, prodromal).

Search terms for PubMed were: (“Lewy Bodies”[Mesh] OR “Lewy Body Disease”[Mesh] OR “Parkinson Disease”[Mesh] OR “Synucleinopathies”[Mesh] OR “alpha-Synuclein”[Mesh]) AND (“Dementia”[Mesh] OR “Cognition Disorders”[Mesh]) AND (“Risk”[Mesh] OR “Prodromal Symptoms”[Mesh]). Search terms for Embase were: (‘lewy bodies’:ti,ab,kw OR ‘lewy body disease’:ti,ab,kw OR ‘parkinson’:ti,ab,kw OR ‘synucleins’:ti,ab,kw OR ‘alpha-synuclein’:ti,ab,kw) AND (‘dementia’:ti,ab,kw OR ‘cognitive decline’:ti,ab,kw OR ‘cognitive impairment’:ti,ab,kw OR ‘mci’:ti,ab,kw) AND (‘risk’:ti,ab,kw OR ‘prodromal symptoms’:ti,ab,kw OR ‘prediction’:ti,ab,kw) AND [english]/lim. Search terms for Web of Science were: ((Lewy OR Parkinson OR synuclein) AND (dementia OR cognitive decline OR cognitive impairment OR MCI) AND (risk OR prodromal OR prediction)).

### Study selection

To include all published original articles with LBD as an outcome in a longitudinal study, the following inclusion and exclusion criteria were applied.

Inclusion criteria:

1. The study had a longitudinal design
2. For clinically defined cohorts, the study included participants without a dementia diagnosis at baseline develop (a) DLB, (b) PDD, or (c) LBD without specification of DLB or PDD during follow-up.
3. For pathologically defined cohorts, the study included participants without a dementia diagnosis at baseline develop dementia during the study period with underlying Lewy body pathology.
4. A risk factor, predictor or prodromal feature is assessed by statistical analysis.

Exclusion criteria:

1. Study focusing on participants without dementia by the end of the study follow-up period (cognitive impairment without specification of dementia, MCI without progression to dementia)
2. Study focusing on participants with dementia at baseline
3. DLB, PDD or LBD not included as an outcome for risk/prediction analysis
4. DLB, PDD, or LBD not assessed on its own but combined with another type of disease or dementia as an outcome
5. Study without a non-exposed group to include in risk/prediction analysis

**Fig. 3:**
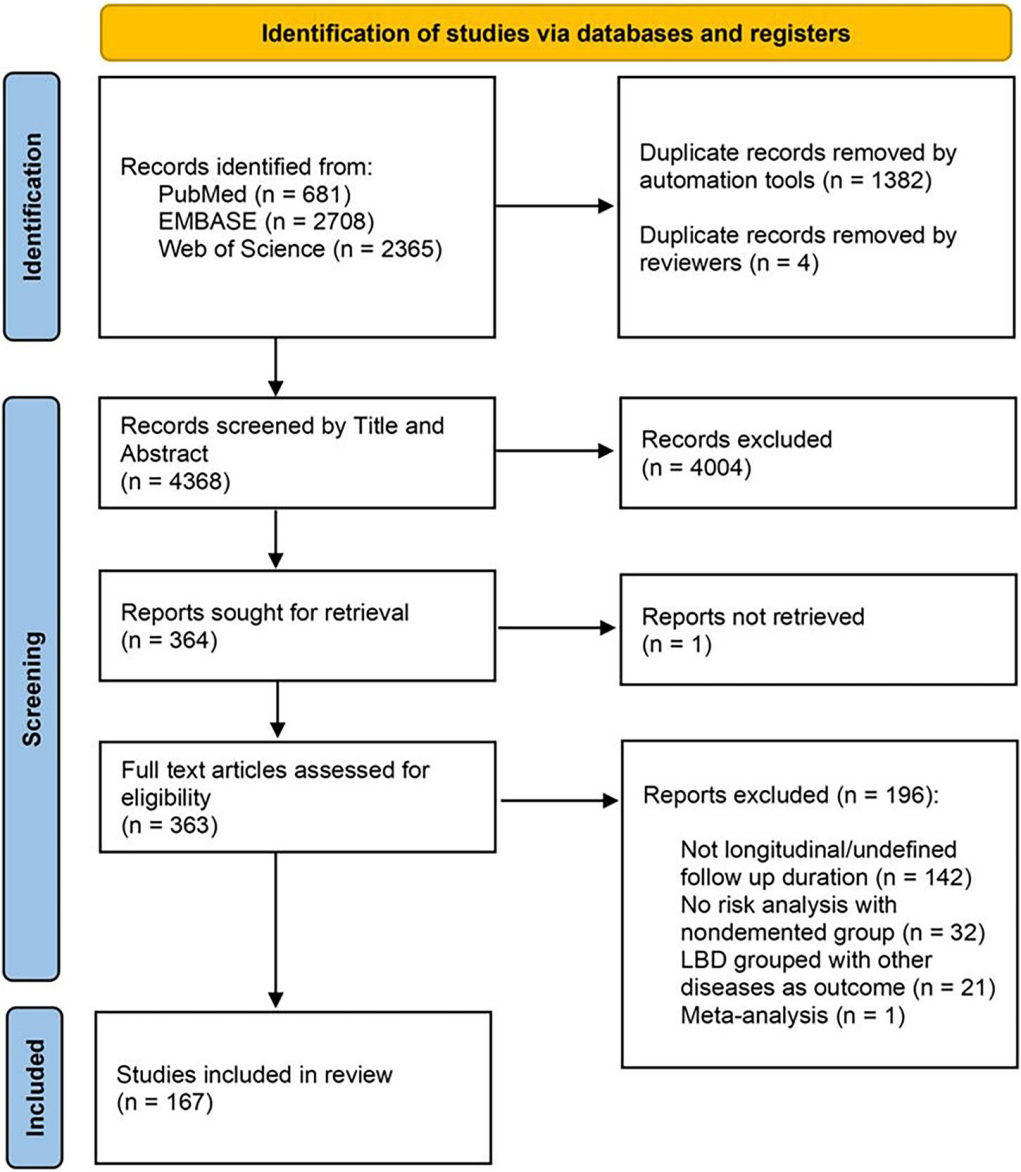
Preferred Reporting Items for Systematic Reviews and Meta-Analyses (PRISMA) diagram for studies in the systematic review.

The search resulted in 681 studies from PubMed, 2708 studies from Embase and 2365 studies from Web of Science (Fig. 3). After removing the duplicate records, a combination of two reviewers (AR, CJ, EB, SA) independently screened the 4368 studies by title and abstract. The selected 364 studies from this step were sought for full text retrieval. One article was obtained after contact with the corresponding author, and one article could not be accessed. Thus, 363 articles were further screened by full text and a combination of two reviewers (AR, CJ, FD, EB, SA) based on the stated inclusion/exclusion criteria. For any disagreement between the two reviewers, a third reviewer screened the studies to resolve the conflict. Overall, 167 studies were included for data extraction and synthesis.

### Data extraction and synthesis

For included studies, data including year of publication, country of study, sample size for risk/predictor analysis, length of follow-up, percent females, mean age at baseline, diagnostic criteria for LBD, and findings on the association between LBD and risk factors were extracted. For consistency, median (range) values were transformed to mean (standard deviation). Each data extraction table was completed by one of four reviewers (AR, FD, EB, SA). Risk factors and predictors were grouped as: non-modifiable (demographic, genetic), modifiable (health condition, medication, environment), and clinical (clinical signs and symptoms, imaging, fluid biomarker) factors. The findings were reported using a narrative synthesis. Since sex and gender terms were used interchangeably in the publications, we grouped sex and gender as sex, with female/male categories for brevity.

### Quality assessment

The quality and risk of bias for the included studies was evaluated with the Newcastle-Ottawa Quality Assessment Scale including a total of 8 items in the domains of selection, comparability, and outcome^208^. Selection includes items assessing whether (1) the exposed cohort (DLB, PDD, or LBD for our review) is truly or somewhat representative of the average affected people (DLB, PDD, or LBD for our review) in the community, (2) the non-exposed cohort (controls without DLB, PDD, or LBD for our review) was selected from the same community as the exposed cohort, (3) exposure (DLB, PDD, or LBD diagnosis) was based on secure record or structured interview, and (4) outcome of interest (DLB, PDD, or LBD diagnosis) was not present at start of study. Comparability questions whether the study controls for the most important factor (age for our review) and any additional factors. Outcome includes items on whether (1) the outcome was assessed by independent blind assessment/record linkage, (2) the follow-up was long enough for outcome to occur (one year for our review), and (3) all subjects were accounted for (complete follow-up), the subjects lost to follow-up were not likely to introduce bias or description was provided of those lost. Total scores on the scale range from 0-2 for poor quality, 3–5 for fair quality and 6-9 for good/high quality. Studies with only good/high quality were included.

## Supplementary information

PRISMA_2020_checklist_9jun25

supplementary tables_lbd risk sysrev

## Data Availability

No datasets were generated or analysed during the current study.

## References

[1] Galvin, J. E. Lewy body dementia. *CONTINUUM: Lifelong Learn. Neurol.* **30**, 1673–1698 (2024). 10.1212/CON.0000000000001496 PMC12094501 39620839

[2] Aarsland, D. & Kurz, M. W. The epidemiology of dementia associated with Parkinson disease. *J. Neurol. Sci.* **289**, 18–22 (2010). 19733364 10.1016/j.jns.2009.08.034

[3] Dorsey, E. R. et al. Global, regional, and national burden of Parkinson’s disease, 1990–2016: a systematic analysis for the Global Burden of Disease Study 2016. *Lancet Neurol.* **17**, 939–953 (2018). 30287051 10.1016/S1474-4422(18)30295-3 PMC6191528

[4] Vann Jones, S. A. & O’Brien, J. T. The prevalence and incidence of dementia with Lewy bodies: a systematic review of population and clinical studies. *Psychol. Med.* **44**, 673–683 (2014). 23521899 10.1017/S0033291713000494

[5] Aarsland, D., Zaccai, J. & Brayne, C. A systematic review of prevalence studies of dementia in Parkinson’s disease. *Mov. Disord.* **20**, 1255–1263 (2005). 16041803 10.1002/mds.20527

[6] Desai, U. et al. Epidemiology and economic burden of Lewy body dementia in the United States. *Curr. Med. Res. Opin.* **38**, 1177–1188 (2022). 35442134 10.1080/03007995.2022.2059978

[7] Emre, M. et al. Clinical diagnostic criteria for dementia associated with Parkinson’s disease. *Mov. Disord.* **22**, 1689–1707 (2007). 17542011 10.1002/mds.21507

[8] McKeith, I. G. et al. Diagnosis and management of dementia with Lewy bodies. *Neurology* **89**, 88–100 (2017). 28592453 10.1212/WNL.0000000000004058 PMC5496518

[9] Holden, S. K. et al. Research priorities of individuals and caregivers with Lewy body dementia. *Alzheimer Dis. Assoc. Disord.* **37**, 50–58 (2023). 36821177 10.1097/WAD.0000000000000545 PMC9971616

[10] Agarwal, K. et al. Lewy body dementia: overcoming barriers and identifying solutions. *Alzheimer’s. Dement.* **20**, 2298–2308 (2024). 38265159 10.1002/alz.13674 PMC10942666

[11] Wang, X. et al. Predictive models of Alzheimer’s disease dementia risk in older adults with mild cognitive impairment: a systematic review and critical appraisal. *BMC Geriatr.* **24**, 531 (2024). 38898411 10.1186/s12877-024-05044-8 PMC11188292

[12] Brain, J. et al. What’s new in dementia risk prediction modelling? An updated systematic review. *Dement Geriatr. Cognit. Dis. Extra* **14**, 49–74 (2024). 39015518 10.1159/000539744 PMC11250535

[13] Wu, Y.-H., Lee, W.-J., Chen, Y.-H., Chang, M.-H. & Lin, C.-H. Premotor symptoms as predictors of outcome in Parkinsons disease: a case-control study. *PLoS ONE* **11**, e0161271 (2016). 27533053 10.1371/journal.pone.0161271 PMC4988705

[14] Bohn, L. et al. Dementia risk prediction in a longitudinal geriatric Parkinson’s disease cohort: evaluation and application of the montreal parkinson risk of dementia scale. *Can. Geriatr. J.* **26**, 176–186 (2023). 36865405 10.5770/cgj.26.617 PMC9953498

[15] Anang, J. B. M. et al. Dementia Predictors in Parkinson disease: a validation study. *J. Parkinsons Dis.* **7**, 159–162 (2017). 27911340 10.3233/JPD-160925

[16] Hughes, T. A. et al. A 10-year study of the incidence of and factors predicting dementia in Parkinson’s disease. *Neurology* **54**, 1596–1603 (2000). 10762499 10.1212/wnl.54.8.1596

[17] Rana, A. Q., Yousuf, M. S., Naz, S. & Qa’aty, N. Prevalence and relation of dementia to various factors in Parkinson’s disease. *Psychiatry Clin. Neurosci.* **66**, 64–68 (2012). 22250611 10.1111/j.1440-1819.2011.02291.x

[18] Zhu, K., van Hilten, J. J. & Marinus, J. Predictors of dementia in Parkinson’s disease; findings from a 5-year prospective study using the SCOPA-COG. *Parkinsonism Relat. Disord.* **20**, 980–985 (2014). 25024059 10.1016/j.parkreldis.2014.06.006

[19] Levy, G. et al. Combined effect of age and severity on the risk of dementia in Parkinson’s disease. *Ann. Neurol.* **51**, 722–729 (2002). 12112078 10.1002/ana.10219

[20] Hindle, J. V. et al. The effects of cognitive reserve and lifestyle on cognition and dementia in Parkinson’s disease—a longitudinal cohort study. *Int. J. Geriatr. Psychiatry* **31**, 13–23 (2016). 25781584 10.1002/gps.4284

[21] Hoogland, J. et al. Mild cognitive impairment as a risk factor for Parkinson’s disease dementia. *Mov. Disord.* **32**, 1056–1065 (2017). 28605056 10.1002/mds.27002

[22] Evans, J. R. et al. The natural history of treated Parkinson’s disease in an incident, community based cohort. *J. Neurol. Neurosurg. Psychiatry* **82**, 1112–1118 (2011). 21593513 10.1136/jnnp.2011.240366

[23] Alves, G. et al. CSF Aβ _42_ predicts early-onset dementia in Parkinson disease. *Neurology* **82**, 1784–1790 (2014). 24748671 10.1212/WNL.0000000000000425

[24] Choi, M. H., Yoon, J. H. & Yong, S. W. Cardiac sympathetic denervation and dementia in de novo Parkinson’s disease: a 7-year follow-up study. *J. Neurol. Sci.* **381**, 291–295 (2017). 28991700 10.1016/j.jns.2017.09.010

[25] Aarsland, D. et al. Risk of dementia in Parkinson’s disease: a community-based, prospective study. *Neurology* **56**, 730–736 (2001). 11274306 10.1212/wnl.56.6.730

[26] Aarsland, D. et al. The effect of age of onset of PD on risk of dementia. *J. Neurol.* **254**, 38–45 (2007). 17508138 10.1007/s00415-006-0234-8

[27] Galtier, I., Nieto, A., Mata, M., Lorenzo, J. N. & Barroso, J. Analyses of visuospatial and visuoperceptual errors as predictors of dementia in Parkinson’s disease patients with subjective cognitive decline and mild cognitive impairment. *J. Int. Neuropsychol. Soc.* **27**, 722–732 (2021). 33303048 10.1017/S1355617720001216

[28] Fitts, W. et al. Caregiver report of apathy predicts dementia in Parkinson’s disease. *Parkinsonism Relat. Disord.* **21**, 992–995 (2015). 26117435 10.1016/j.parkreldis.2015.06.009 PMC4509809

[29] Horne, K. et al. Neuropsychiatric symptoms are associated with dementia in Parkinson’s disease but not predictive of it. *Mov. Disord. Clin. Pract.* **8**, 390–399 (2021). 33816668 10.1002/mdc3.13151 PMC8015884

[30] Jacobs, D. M. et al. Neuropsychological characteristics of preclinical dementia in Parkinson’s disease. *Neurology* **45**, 1691–1696 (1995). 7675228 10.1212/wnl.45.9.1691

[31] Nomura, T., Inoue, Y., Kagimura, T. & Nakashima, K. Clinical significance of REM sleep behavior disorder in Parkinson’s disease. *Sleep. Med.* **14**, 131–135 (2013). 23218532 10.1016/j.sleep.2012.10.011

[32] Park, C. J. et al. An interpretable multiparametric radiomics model of basal ganglia to predict dementia conversion in Parkinson’s disease. *NPJ Parkinsons Dis.* **9**, 127 (2023). 37648733 10.1038/s41531-023-00566-1 PMC10468504

[33] Stern, Y., Marder, K., Tang, M. X. & Mayeux, R. Antecedent clinical features associated with dementia in Parkinson’s disease. *Neurology* **43**, 1690–1690 (1993). 8414013 10.1212/wnl.43.9.1690

[34] McFall, G. P. et al. Identifying key multi-modal predictors of incipient dementia in Parkinson’s disease: a machine learning analysis and Tree SHAP interpretation. *Front. Aging Neurosci.* **15**, 1124232 (2023). 10.3389/fnagi.2023.1124232 PMC10347530 37455938

[35] Williams-Gray, C. H. et al. The distinct cognitive syndromes of Parkinson’s disease: 5 year follow-up of the CamPaIGN cohort. *Brain* **132**, 2958–2969 (2009). 19812213 10.1093/brain/awp245

[36] Hoogland, J. et al. Risk of Parkinson’s disease dementia related to level I MDS PD-MCI. *Mov. Disord.* **34**, 430–435 (2019). 30653248 10.1002/mds.27617

[37] Kwon, K.-Y. et al. Nonmotor symptoms and cognitive decline in de novo Parkinson’s disease. *Can. J. Neurol. Sci.* **41**, 597–602 (2014). 25373810 10.1017/cjn.2014.3

[38] Bakeberg, M. C. et al. Differential effects of sex on longitudinal patterns of cognitive decline in Parkinson’s disease. *J. Neurol.* **268**, 1903–1912 (2021). 33399968 10.1007/s00415-020-10367-8 PMC8068663

[39] Fink, A., Dodel, R., Georges, D. & Doblhammer, G. The impact of sex-specific survival on the incidence of dementia in Parkinson’s disease. *Mov. Disord.* **38**, 2041–2052 (2023). 37658585 10.1002/mds.29596

[40] Phongpreecha, T. et al. Multivariate prediction of dementia in Parkinson’s disease. *NPJ Parkinsons Dis.* **6**, 20 (2020). 32885039 10.1038/s41531-020-00121-2 PMC7447766

[41] Jeong, S. H., Lee, H. S., Lee, P. H., Sohn, Y. H. & Chung, S. J. Does dopamine deficiency affect sex-dependent prognosis in Parkinson’s disease?. *Parkinsonism Relat. Disord.* **102**, 57–63 (2022). 35961198 10.1016/j.parkreldis.2022.07.012

[42] Breteler, M. M. B., de Groot, R. R. M., van Romunde, L. K. J. & Hofman, A. Risk of dementia in patients with Parkinson’s disease, epilepsy, and severe head trauma: a register-based follow-up study. *Am. J. Epidemiol.* **142**, 1300–1305 (1995). 7503050 10.1093/oxfordjournals.aje.a117597

[43] Hobson, P. & Meara, J. Risk and incidence of dementia in a cohort of older subjects with Parkinson’s disease in the United Kingdom. *Mov. Disord.* **19**, 1043–1049 (2004). 15372593 10.1002/mds.20216

[44] Umeh, C. C., Mahajan, A., Mihailovic, A. & Pontone, G. M. APOE4 allele, sex, and dementia Risk in Parkinson’s disease: lessons from a longitudinal cohort. *J. Geriatr. Psychiatry Neurol.* **35**, 810–815 (2022). 34958617 10.1177/08919887211060019 PMC11062588

[45] Counsell, C., Giuntoli, C., Khan, Q. I., Maple-Grødem, J. & Macleod, A. D. The incidence, baseline predictors, and outcomes of dementia in an incident cohort of Parkinson’s disease and controls. *J. Neurol.* **269**, 4288–4298 (2022). 35307754 10.1007/s00415-022-11058-2 PMC9294013

[46] Winder-Rhodes, S. E. et al. Glucocerebrosidase mutations influence the natural history of Parkinson’s disease in a community-based incident cohort. *Brain* **136**, 392–399 (2013). 23413260 10.1093/brain/aws318

[47] Cilia, R. et al. Survival and dementia in GBA-associated Parkinson’s disease: the mutation matters. *Ann. Neurol.* **80**, 662–673 (2016). 27632223 10.1002/ana.24777

[48] Liu, G. et al. Prediction of cognition in Parkinson’s disease with a clinical–genetic score: a longitudinal analysis of nine cohorts. *Lancet Neurol.* **16**, 620–629 (2017). 28629879 10.1016/S1474-4422(17)30122-9 PMC5761650

[49] Lunde, K. A. et al. Association of glucocerebrosidase polymorphisms and mutations with dementia in incident Parkinson’s disease. *Alzheimer’s. Dement.* **14**, 1293–1301 (2018). 29792872 10.1016/j.jalz.2018.04.006

[50] Stoker, T. B. et al. Impact of *GBA1* variants on long-term clinical progression and mortality in incident Parkinson’s disease. *J. Neurol. Neurosurg. Psychiatry* **91**, 695–702 (2020). 32303560 10.1136/jnnp-2020-322857 PMC7361014

[51] Szwedo, A. A. et al. GBA and APOE impact cognitive decline in Parkinson’s disease: a 10-Year population-based study. *Mov. Disord.* **37**, 1016–1027 (2022). 35106798 10.1002/mds.28932 PMC9362732

[52] Liu, G. et al. Genome-wide survival study identifies a novel synaptic locus and polygenic score for cognitive progression in Parkinson’s disease. *Nat. Genet.* **53**, 787–793 (2021). 33958783 10.1038/s41588-021-00847-6 PMC8459648

[53] Vijiaratnam, N. et al. Combining biomarkers for prognostic modelling of Parkinson’s disease. *J. Neurol. Neurosurg. Psychiatry* **93**, 707–715 (2022). 35577512 10.1136/jnnp-2021-328365 PMC9279845

[54] Kurz, M. W. et al. APOE Alleles in Parkinson disease and their relationship to cognitive decline: a population-based, longitudinal study. *J. Geriatr. Psychiatry Neurol.* **22**, 166–170 (2009). 19321880 10.1177/0891988709332945

[55] Myers, P. S. et al. Proteinopathy and longitudinal cognitive decline in Parkinson disease. *Neurology* **99**, e66–e76 (2022). 10.1212/WNL.0000000000200344 PMC9259093 35418463

[56] Goris, A. et al. Tau and α-synuclein in susceptibility to, and dementia in, Parkinson’s disease. *Ann. Neurol.* **62**, 145–153 (2007). 17683088 10.1002/ana.21192

[57] Lin, C.-H., Fan, J.-Y., Lin, H.-I., Chang, C.-W. & Wu, Y.-R. Catechol-O-methyltransferase (COMT) genetic variants are associated with cognitive decline in patients with Parkinson’s disease. *Parkinsonism Relat. Disord.* **50**, 48–53 (2018). 29439855 10.1016/j.parkreldis.2018.02.015

[58] Bäckström, D. et al. Polymorphisms in dopamine-associated genes and cognitive decline in Parkinson’s disease. *Acta Neurol. Scand.* **137**, 91–98 (2018). 28869277 10.1111/ane.12812 PMC5763317

[59] Degerman, S. et al. Long leukocyte telomere length at diagnosis is a risk factor for dementia progression in idiopathic Parkinsonism. *PLoS ONE* **9**, e113387 (2014). 25501556 10.1371/journal.pone.0113387 PMC4264694

[60] Bäckström, D. et al. PITX3 genotype and risk of dementia in Parkinson’s disease: a population-based study. *J. Neurol. Sci.* **381**, 278–284 (2017). 28991698 10.1016/j.jns.2017.08.3259

[61] Corrado, L. et al. The length of SNCA Rep1 microsatellite may influence cognitive evolution in Parkinson’s disease. *Front. Neurol.* **9**, 213 (2018). 10.3389/fneur.2018.00213 PMC5890103 29662465

[62] Fang, Y. et al. Aquaporin-4 polymorphisms are associated with cognitive performance in Parkinson’s disease. *Front. Aging Neurosci.* **13**, 740491 (2022). 10.3389/fnagi.2021.740491 PMC8959914 35356146

[63] Bakeberg, M. C. et al. TOMM40 ‘523’ poly-T repeat length is a determinant of longitudinal cognitive decline in Parkinson’s disease. *NPJ Parkinsons Dis.* **7**, 56 (2021). 34234128 10.1038/s41531-021-00200-y PMC8263775

[64] Tunold, J. et al. Lysosomal polygenic burden drives cognitive decline in Parkinson’s disease with lw Alzheimer risk. *Mov. Disord.* **39**, 596–601 (2024). 38124396 10.1002/mds.29698

[65] Lee, S.-Y. et al. Dementia-free survival and risk factors for dementia in a hospital-Based Korean Parkinson’s disease cohort. *J. Clin. Neurol.* **13**, 21 (2017). 27730764 10.3988/jcn.2017.13.1.21 PMC5242167

[66] Borda, M. G. et al. Frailty in Parkinson’s disease and its association with early dementia: a longitudinal study. *Parkinsonism Relat. Disord.* **99**, 51–57 (2022). 35598420 10.1016/j.parkreldis.2022.05.004

[67] Ebmeier, K. P. et al. Clinical features predicting dementia in idiopathic Parkinson’s disease. *Neurology* **40**, 1222–1222 (1990). 2381529 10.1212/wnl.40.8.1222

[68] Levy, G. et al. Do risk factors for Alzheimer’s disease predict dementia in Parkinson’s disease? an exploratory study. *Mov. Disord.* **17**, 250–257 (2002). 11921109 10.1002/mds.10086

[69] Leta, V. et al. Constipation is associated with development of cognitive impairment in de novo Parkinson’s disease: a longitudinal analysis of two international cohorts. *J. Parkinsons Dis.* **11**, 1209–1219 (2021). 33843697 10.3233/JPD-212570

[70] Jurcau, A. & Nunkoo, V. S. Clinical markers may identify patients at risk for early Parkinson’s disease dementia: a prospective study. *Am. J. Alzheimers Dis. Other Demen.* **36**, 15333175211021369 (2021). 10.1177/15333175211021369 PMC10624063 34075800

[71] Anang, J. B. M. et al. Predictors of dementia in Parkinson disease: a prospective cohort study. *Neurology* **83**, 1253–1260 (2014). 25171928 10.1212/WNL.0000000000000842 PMC4180482

[72] Postuma, R. B. et al. Rapid eye movement sleep behavior disorder and risk of dementia in Parkinson’s disease: a prospective study. *Mov. Disord.* **27**, 720–726 (2012). 22322798 10.1002/mds.24939

[73] Zhong, R., Gan, C., Sun, H. & Zhang, K. Sleep disturbances, cognitive decline, and AD biomarkers alterations in early Parkinson’s disease. *Ann. Clin. Transl. Neurol.* **11**, 1831–1839 (2024). 38764318 10.1002/acn3.52089 PMC11251484

[74] Tajiri, Y. et al. A Single-institution study on predictors of short-term progression from mild cognitive impairment in Parkinson’s disease to Parkinson’s disease with dementia. *Yonago Acta Med*. **63**, 28–33 (2020). 32158330 10.33160/yam.2020.02.004 PMC7028534

[75] Longardner, K. et al. Differential impact of individual autonomic domains on clinical outcomes in Parkinson’s disease. *J. Neurol.* **269**, 5510–5520 (2022). 35708788 10.1007/s00415-022-11221-9 PMC9201260

[76] Kang, S. H. et al. Independent effect of cardiometabolic syndromes and depression on dementia in Parkinson’s disease: a 12-year longitudinal follow-up study of a nationwide cohort. *Eur. J. Neurol.* **30**, 911–919 (2023). 36692249 10.1111/ene.15689

[77] Liang, Y.-T., Lin, C.-Y., Wang, Y.-H., Chou, H.-H. & Wei, J. C.-C. Associations of Chinese herbal medicine usage with risk of dementia in patients with Parkinson’s disease: a population-based, nested case–control study. *J. Alternative Complementary Med.* **27**, 606–612 (2021). 10.1089/acm.2020.0422 33979532

[78] Onofrj, M. et al. Preexisting bipolar disorder influences the subsequent phenotype of Parkinson’s Disease. *Mov. Disord.* **36**, 2840–2852 (2021). 34427338 10.1002/mds.28745 PMC9292484

[79] Gerakios, F. et al. Delirium is more common and associated with worse outcomes in Parkinson’s disease compared to older adult controls: results of two prospective longitudinal cohort studies. *Age Ageing* **53**, afae046 (2024). 10.1093/ageing/afae046 PMC10945294 38497236

[80] Baba, T. et al. Severe olfactory dysfunction is a prodromal symptom of dementia associated with Parkinson’s disease: a 3 year longitudinal study. *Brain* **135**, 161–169 (2012). 22287381 10.1093/brain/awr321

[81] Bäckström, D. et al. Prediction and early biomarkers of cognitive decline in Parkinson disease and atypical parkinsonism: a population-based study. *Brain Commun* **4**, fcac040 (2022). 10.1093/braincomms/fcac040 PMC8947320 35350553

[82] Domellöf, M. E., Lundin, K.-F., Edström, M. & Forsgren, L. Olfactory dysfunction and dementia in newly diagnosed patients with Parkinson’s disease. *Parkinsonism Relat. Disord.* **38**, 41–47 (2017). 28242255 10.1016/j.parkreldis.2017.02.017

[83] Campos-Sousa, R. N. et al. Longitudinal analysis of functional disabilities, cognitive decline and risk of dementia in women with Parkinson’s disease and detrusor overactivity. *J. Clin. Neurosci.* **75**, 85–88 (2020). 32245601 10.1016/j.jocn.2020.03.019

[84] Kang, S. H. et al. Fasting glucose variability and risk of dementia in Parkinson’s disease: a 9-year longitudinal follow-up study of a nationwide cohort. *Front. Aging Neurosci.* **15**, 1292524 (2024). 10.3389/fnagi.2023.1292524 PMC10791804 38235038

[85] Peng, Z. et al. Metabolic syndrome contributes to cognitive impairment in patients with Parkinson’s disease. *Parkinsonism Relat. Disord.* **55**, 68–74 (2018). 29908727 10.1016/j.parkreldis.2018.05.013

[86] Aarsland, D., Andersen, K., Larsen, J. P. & Lolk, A. Prevalence and characteristics of dementia in Parkinson disease. *Arch. Neurol.* **60**, 387 (2003). 12633150 10.1001/archneur.60.3.387

[87] Bugalho, P. & Viana-Baptista, M. Predictors of cognitive decline in the early stages of Parkinson’s disease: a brief cognitive assessment longitudinal study. *Parkinsons Dis.* **2013**, 1–8 (2013). 10.1155/2013/912037 PMC3835472 24303226

[88] Gryc, W. et al. Hallucinations and development of dementia in Parkinson’s disease. *J. Parkinsons Dis.* **10**, 1643–1648 (2020). 32741842 10.3233/JPD-202116 PMC7609584

[89] Bejr-kasem, H. et al. Minor hallucinations reflect early gray matter loss and predict subjective cognitive decline in Parkinson’s disease. *Eur. J. Neurol.* **28**, 438–447 (2021). 33032389 10.1111/ene.14576

[90] Ramirez-Ruiz, B., Junque, C., Marti, M.-J., Valldeoriola, F. & Tolosa, E. Cognitive changes in Parkinson’s disease patients with visual hallucinations. *Dement Geriatr. Cognit. Disord.* **23**, 281–288 (2007). 17351320 10.1159/000100850

[91] Sanyal, J., Banerjee, T. K. & Rao, V. R. Dementia and Cognitive impairment in patients With Parkinson’s disease from India. *Am. J. Alzheimers Dis. Other Demen.* **29**, 630–636 (2014). 24771763 10.1177/1533317514531442 PMC10852774

[92] Lee, Y. et al. Association of neuropsychiatric symptom profiles with cognitive decline in patients with Parkinson disease and mild cognitive impairment. *Neurology* **101**, e1186–e1195 (2023). 10.1212/WNL.0000000000207623 PMC10516268 37524535

[93] Haugarvoll, K., Aarsland, D., Wentzel-Larsen, T. & Larsen, J. P. The influence of cerebrovascular risk factors on incident dementia in patients with Parkinson’s disease. *Acta Neurol. Scand.* **112**, 386–390 (2005). 16281921 10.1111/j.1600-0404.2005.00389.x

[94] Qu, Y. et al. Estimated glomerular filtration rate is a biomarker of cognitive impairment in Parkinson’s disease. *Front. Aging Neurosci.* **15**, 1130833 (2023). 10.3389/fnagi.2023.1130833 PMC10240071 37284018

[95] Yoo, H. S., Chung, S. J., Lee, P. H., Sohn, Y. H. & Kang, S. Y. The influence of body mass index at diagnosis on cognitive decline in Parkinson’s disease. *J. Clin. Neurol.* **15**, 517 (2019). 31591841 10.3988/jcn.2019.15.4.517 PMC6785479

[96] Jung, J. H. et al. Effects of dihydropyridines on the motor and cognitive outcomes of patients with Parkinson’s disease. *Mov. Disord.* **38**, 843–853 (2023). 36825772 10.1002/mds.29367

[97] Jeong, S. H. et al. Effects of statins on dopamine loss and prognosis in Parkinson’s disease. *Brain* **144**, 3191–3200 (2021). 34347020 10.1093/brain/awab292

[98] Sheu, J., Tsai, M., Erickson, S. R. & Wu, C. Association between anticholinergic medication use and risk of dementia among patients with Parkinson’s disease. *Pharmacotherapy: J. Hum. Pharmacol. Drug Ther.* **39**, 798–808 (2019). 10.1002/phar.2305 31251824

[99] Hong, C.-T., Chan, L., Wu, D., Chen, W.-T. & Chien, L.-N. Antiparkinsonism anticholinergics increase dementia risk in patients with Parkinson’s disease. *Parkinsonism Relat. Disord.* **65**, 224–229 (2019). 31255536 10.1016/j.parkreldis.2019.06.022

[100] Yoo, H. S. et al. Levodopa-induced dyskinesia is closely linked to progression of frontal dysfunction in PD. *Neurology* **92**, e1468–e1478 (2019). 10.1212/WNL.0000000000007189 30796137

[101] Becker, S. et al. Cognitive-driven activities of daily living impairment as a predictor for dementia in Parkinson disease. *Neurology* **99**, e2548–e2560 (2022). 10.1212/WNL.0000000000201201 PMC9754648 36240089

[102] Boel, J. A. et al. Level I PD-MCI using Global Cognitive Tests and the risk for Parkinson’s disease dementia. *Mov. Disord. Clin. Pract.* **9**, 479–483 (2022). 35582313 10.1002/mdc3.13451 PMC9092740

[103] Campbell, M. C. et al. Parkinson disease clinical subtypes: key features & clinical milestones. *Ann. Clin. Transl. Neurol.* **7**, 1272–1283 (2020). 32602253 10.1002/acn3.51102 PMC7448190

[104] Domellöf, M. E., Ekman, U., Forsgren, L. & Elgh, E. Cognitive function in the early phase of Parkinson’s disease, a five-year follow-up. *Acta Neurol. Scand.* **132**, 79–88 (2015). 25644230 10.1111/ane.12375

[105] Pedersen, K. F., Larsen, J. P., Tysnes, O.-B. & Alves, G. Natural course of mild cognitive impairment in Parkinson disease. *Neurology* **88**, 767–774 (2017). 28108638 10.1212/WNL.0000000000003634

[106] Pedersen, K. F., Larsen, J. P., Tysnes, O.-B. & Alves, G. Prognosis of mild cognitive impairment in early Parkinson disease. *JAMA Neurol.* **70**, 580 (2013). 23529397 10.1001/jamaneurol.2013.2110

[107] Nicoletti, A. et al. Incidence of mild cognitive impairment and dementia in Parkinson’s disease: the Parkinson’s disease cognitive impairment study. *Front. Aging Neurosci.* **11**, 21 (2019). 10.3389/fnagi.2019.00021 PMC6376919 30800065

[108] Nicoletti, A. et al. Vascular risk factors, white matter lesions and cognitive impairment in Parkinson’s disease: the PACOS longitudinal study. *J. Neurol.* **268**, 549–558 (2021). 32865628 10.1007/s00415-020-10189-8 PMC7880923

[109] De Roy, J. et al. Detecting the cognitive prodrome of dementia in Parkinson’s disease. *J. Parkinsons Dis.* **10**, 1033–1046 (2020). 32310188 10.3233/JPD-191857

[110] Gasca-Salas, C. et al. Longitudinal assessment of the pattern of cognitive decline in non-demented patients with advanced Parkinson’s disease. *J. Parkinsons Dis.* **4**, 677–686 (2014). 25208730 10.3233/JPD-140398

[111] Janvin, C. C., Larsen, J. P., Aarsland, D. & Hugdahl, K. Subtypes of mild cognitive impairment in parkinson’s disease: progression to dementia. *Mov. Disord.* **21**, 1343–1349 (2006). 16721732 10.1002/mds.20974

[112] Chung, S. J. et al. Clinical relevance of amnestic versus non-amnestic mild cognitive impairment subtyping in Parkinson’s disease. *Eur. J. Neurol.* **26**, 766–773 (2019). 30565368 10.1111/ene.13886

[113] Kurlan, R. et al. Early clinical predictors of treatment-resistant and functional outcomes in Parkinson’s disease. *Mov. Disord. Clin. Pract.* **3**, 53–58 (2016). 30363507 10.1002/mdc3.12273 PMC6178710

[114] Jalakas, M. et al. A quick test of cognitive speed can predict development of dementia in Parkinson’s disease. *Sci. Rep.* **9**, 15417 (2019). 31659172 10.1038/s41598-019-51505-1 PMC6817840

[115] Ye, B. S. et al. Dementia-predicting cognitive risk score and its correlation with cortical thickness in Parkinson disease. *Dement Geriatr. Cognit. Disord.* **44**, 203–212 (2017). 28930751 10.1159/000479057

[116] Olde Dubbelink, K. T. E. et al. Predicting dementia in Parkinson disease by combining neurophysiologic and cognitive markers. *Neurology* **82**, 263–270 (2014). 24353335 10.1212/WNL.0000000000000034

[117] Lee, J. E. et al. Exploratory analysis of neuropsychological and neuroanatomical correlates of progressive mild cognitive impairment in Parkinson’s disease. *J. Neurol. Neurosurg. Psychiatry* **85**, 7–16 (2014). 23828835 10.1136/jnnp-2013-305062

[118] Mahieux, F. et al. Neuropsychological prediction of dementia in Parkinson’s disease. *J. Neurol. Neurosurg. Psychiatry* **64**, 178–183 (1998). 9489527 10.1136/jnnp.64.2.178 PMC2169963

[119] Chung, S. J. et al. Factor analysis–derived cognitive profile predicting early dementia conversion in PD. *Neurology* **95**, e1650–e1659 (2020). 32651296 10.1212/WNL.0000000000010347

[120] Galtier, I., Nieto, A., Mata, M., Lorenzo, J. N. & Barroso, J. Specific pattern of linguistic impairment in Parkinson’s disease patients with subjective cognitive decline and mild cognitive impairment predicts dementia. *J. Int. Neuropsycho. Soc.* **29**, 632–640 (2023). 10.1017/S1355617722000571 36226685

[121] Summers, D., Spencer, K., Okasaki, C. & Huber, J. E. An examination of cognitive heterogeneity in Parkinson disease: the dual-syndrome hypothesis. *J. Speech Lang. Hearing Res.* **67**, 1127–1135 (2024). 10.1044/2024_JSLHR-23-00621 38446552

[122] Compta, Y. et al. Combined dementia-risk biomarkers in Parkinson’s disease: a prospective longitudinal study. *Parkinsonism Relat. Disord.* **19**, 717–724 (2013). 23643469 10.1016/j.parkreldis.2013.03.009

[123] Cholerton, B. et al. Sex differences in progression to mild cognitive impairment and dementia in Parkinson’s disease. *Parkinsonism Relat. Disord.* **50**, 29–36 (2018). 29478836 10.1016/j.parkreldis.2018.02.007 PMC5943177

[124] Massa, F. et al. Neuroimaging findings and clinical trajectories of Lewy body disease in patients with MCI. *Neurobiol. Aging* **76**, 9–17 (2019). 30611093 10.1016/j.neurobiolaging.2018.12.001

[125] Galtier, I., Nieto, A., Lorenzo, J. N. & Barroso, J. Mild cognitive impairment in Parkinsons disease: diagnosis and progression to dementia. *J. Clin. Exp. Neuropsychol.* **38**, 40–50 (2016). 26602176 10.1080/13803395.2015.1087465

[126] Galtier, I., Nieto, A., Lorenzo, J. N. & Barroso, J. Subjective cognitive decline and progression to dementia in Parkinson’s disease: a long-term follow-up study. *J. Neurol.* **266**, 745–754 (2019). 30635723 10.1007/s00415-019-09197-0

[127] Chung, S. J. et al. Potential link between cognition and motor reserve in patients with Parkinson’s disease. *J. Mov. Disord.* **15**, 249–257 (2022). 36065615 10.14802/jmd.22063 PMC9536917

[128] Marder, K., Tang, M.-X., Cote, L., Stern, Y. & Mayeux, R. The frequency and associated risk factors for dementia in patients With Parkinson’s disease. *Arch. Neurol.* **52**, 695–701 (1995). 7619026 10.1001/archneur.1995.00540310069018

[129] Alves, G., Larsen, J. P., Emre, M., Wentzel-Larsen, T. & Aarsland, D. Changes in motor subtype and risk for incident dementia in Parkinson’s disease. *Mov. Disord.* **21**, 1123–1130 (2006). 16637023 10.1002/mds.20897

[130] Burn, D. J. et al. Motor subtype and cognitive decline in Parkinson’s disease, Parkinson’s disease with dementia, and dementia with Lewy bodies. *J. Neurol. Neurosurg. Psychiatry* **77**, 585–589 (2006). 16614017 10.1136/jnnp.2005.081711 PMC2117449

[131] Lee, S.-J. & Lee, D.-G. The cross-sectional and longitudinal relationships between white matter hyperintensities and dementia in patients with Parkinson’s disease: a retrospective analysis of 132 patients in a single center. *Arch. Gerontol. Geriatr.* **62**, 133–137 (2016). 26541556 10.1016/j.archger.2015.10.006

[132] Gago, M. F. et al. How do cognitive and axial motor signs correlate in Parkinson’s disease? A 6-year prospective study. *J. Neurol.* **256**, 1655–1662 (2009). 19471849 10.1007/s00415-009-5174-7

[133] Modreanu, R. et al. Cross-sectional and longitudinal associations of motor fluctuations and non-motor predominance with cerebrospinal τ and Aβ as well as dementia-risk in Parkinson’s disease. *J. Neurol. Sci.* **373**, 223–229 (2017). 28131192 10.1016/j.jns.2016.12.064

[134] González-Redondo, R. et al. The impact of silent vascular brain burden in cognitive impairment in Parkinson’s disease. *Eur. J. Neurol.* **19**, 1100–1107 (2012). 22360775 10.1111/j.1468-1331.2012.03682.x

[135] Jeong, S. H. et al. Association between choroid plexus volume and cognition in Parkinson disease. *Eur. J. Neurol.* **30**, 3114–3123 (2023). 37498202 10.1111/ene.15999

[136] Kandiah, N. et al. Hippocampal volume and white matter disease in the prediction of dementia in Parkinson’s disease. *Parkinsonism Relat. Disord.* **20**, 1203–1208 (2014). 25258331 10.1016/j.parkreldis.2014.08.024

[137] Pereira, J. B. et al. Longitudinal degeneration of the basal forebrain predicts subsequent dementia in Parkinson’s disease. *Neurobiol. Dis.* **139**, 104831 (2020). 32145376 10.1016/j.nbd.2020.104831

[138] de Weerd, A. W., Perquin, W. V. M. & Jonkman, E. J. Role of the EEG in the prediction of dementia in Parkinson’s Disease. *Dement. Geriatr. Cognit. Disord.* **1**, 115–118 (1990).

[139] Latreille, V. et al. Electroencephalographic prodromal markers of dementia across conscious states in Parkinson’s disease. *Brain* **139**, 1189–1199 (2016). 26912643 10.1093/brain/aww018 PMC5841211

[140] Imarisio, A. et al. Atypical brain FDG-PET patterns increase the risk of long-term cognitive and motor progression in Parkinson’s disease. *Parkinsonism Relat. Disord.* **115**, 105848 (2023). 37716228 10.1016/j.parkreldis.2023.105848

[141] Pilotto, A. et al. Single-subject SPM FDG-PET patterns predict risk of dementia progression in Parkinson disease. *Neurology* **90**, e1029–e1037 (2018). 10.1212/WNL.0000000000005161 29453242

[142] Chung, S. J. et al. Patterns of striatal dopamine depletion in early Parkinson disease. *Neurology* **95**, e280–e290 (2020). 10.1212/WNL.0000000000009878 32616674

[143] Löhle, M. et al. Putaminal dopamine turnover in de novo Parkinson’s disease predicts later neuropsychiatric fluctuations but not other major health outcomes. *J. Parkinsons Dis.* **9**, 693–704 (2019). 31381528 10.3233/JPD-191672

[144] Chung, S. J. et al. Is the cingulate island sign a marker for early dementia conversion in Parkinson’s disease?. *Eur. J. Neurol.* **30**, 3732–3740 (2023). 37505994 10.1111/ene.16007

[145] Jeong, S. H. et al. Differential implications of cerebral hypoperfusion and hyperperfusion in Parkinson’s disease. *Mov. Disord.* **38**, 1881–1890 (2023). 37489576 10.1002/mds.29565

[146] Chung, S. J. et al. Patterns of regional cerebral hypoperfusion in early Parkinson’s disease: clinical implications. *Parkinsonism Relat. Disord.* **121**, 106024 (2024). 38377658 10.1016/j.parkreldis.2024.106024

[147] Bäckström, D. C. et al. Cerebrospinal fluid patterns and the risk of future dementia in early, incident Parkinson disease. *JAMA Neurol.* **72**, 1175 (2015). 26258692 10.1001/jamaneurol.2015.1449

[148] Cousins, K. A. Q. et al. Evaluation of ATN _PD_ Framework and Biofluid Markers to Predict Cognitive Decline in Early Parkinson Disease. *Neurology* **102**, e208033 (2024). 10.1212/WNL.0000000000208033 PMC11383879 38306599

[149] Sheng, Z.-H. et al. Cerebrospinal fluid neurofilament dynamic profiles predict cognitive progression in individuals with de novo Parkinson’s disease. *Front. Aging Neurosci.* **14**, 1061096 (2022). 10.3389/fnagi.2022.1061096 PMC9802677 36589544

[150] Liu, T. et al. Cerebrospinal fluid GFAP is a predictive biomarker for conversion to dementia and Alzheimer’s disease-associated biomarkers alterations among de novo Parkinson’s disease patients: a prospective cohort study. *J. Neuroinflamm.* **20**, 167 (2023). 10.1186/s12974-023-02843-5 PMC10357612 37475029

[151] Oftedal, L. et al. Association of CSF glucocerebrosidase activity with the risk of incident dementia in patients with Parkinson Disease. *Neurology* **100**, e388–e395 (2023). 36253102 10.1212/WNL.0000000000201418 PMC9897053

[152] Delgado-Alvarado, M. et al. Ratios of proteins in cerebrospinal fluid in Parkinson’s disease cognitive decline: prospective study. *Mov. Disord.* **33**, 1809–1813 (2018). 30423201 10.1002/mds.27518

[153] Ma, L.-Z. et al. Serum neurofilament dynamics predicts cognitive progression in de novo Parkinson’s disease. *J. Parkinsons Dis.* **11**, 1117–1127 (2021). 33935105 10.3233/JPD-212535

[154] McCarter, S. J. et al. Higher vitamin B12 level at Parkinson’s disease diagnosis is associated with lower risk of future dementia. *Parkinsonism Relat. Disord.* **73**, 19–22 (2020). 32203914 10.1016/j.parkreldis.2020.03.009

[155] Lee, J., Park, S. & Jang, W. Serum zinc deficiency could be associated with dementia conversion in Parkinson’s disease. *Front. Aging Neurosci.* **15**, 1132907 (2023). 37181629 10.3389/fnagi.2023.1132907 PMC10172503

[156] Chen-Plotkin, A. S. et al. Plasma epidermal growth factor levels predict cognitive decline in Parkinson disease. *Ann. Neurol.* **69**, 655–663 (2011). 21520231 10.1002/ana.22271 PMC3155276

[157] McKeith, I. G. et al. Diagnosis and management of dementia with Lewy bodies: third report of the DLB consortium. *Neurology* **65**, 1863–1872 (2005). 16237129 10.1212/01.wnl.0000187889.17253.b1

[158] Shim, Y., Park, M. & Kim, J. Increased incidence of dementia following herpesvirus infection in the Korean population. *Medicine* **101**, e31116 (2022). 36254002 10.1097/MD.0000000000031116 PMC9575754

[159] Golimstok, Á et al. Adult attention deficit-hyperactivity disorder is associated with Lewy body disease and cognitive impairment: a prospective cohort study with 15-year follow-up. *Am. J. Geriatr. Psychiatry* **32**, 1063–1077 (2024). 38697886 10.1016/j.jagp.2024.04.005

[160] Hart, A., Aldridge, G., Zhang, Q., Narayanan, N. S. & Simmering, J. E. Association of Terazosin, Doxazosin, or Alfuzosin use and risk of dementia with Lewy bodies in men. *Neurology* **103**, e209570 (2024). 38896813 10.1212/WNL.0000000000209570 PMC11226317

[161] Iranzo, A. et al. Neurodegenerative disease status and post-mortem pathology in idiopathic rapid-eye-movement sleep behaviour disorder: an observational cohort study. *Lancet Neurol.* **12**, 443–453 (2013). 23562390 10.1016/S1474-4422(13)70056-5

[162] Jung, Y. et al. Phenoconversion from probable rapid eye movement sleep behavior disorder to mild cognitive impairment to dementia in a population-based sample. *Alzheimer’s Dement. Diagnosis Assess. Dis. Monit.* **8**, 127–130 (2017). 10.1016/j.dadm.2017.05.004 PMC5470599 28649596

[163] Zolfaghari, S. et al. Cardiovascular risk factors and phenoconversion to neurodegenerative synucleinopathies in idiopathic REM sleep behavior disorder. *J. Parkinsons Dis.* **12**, 927–933 (2022). 35001898 10.3233/JPD-212984 PMC9789479

[164] Wang, J. et al. Residual injurious symptoms and its association with neurodegenerative outcomes in idiopathic rapid eye movement sleep behavior disorder: a retrospective, longitudinal follow-up study. *Mov. Disord.* **35**, 2077–2085 (2020). 32744735 10.1002/mds.28210

[165] Marchand, D. G., Montplaisir, J., Postuma, R. B., Rahayel, S. & Gagnon, J.-F. Detecting the cognitive prodrome of dementia with Lewy bodies: a prospective study of REM sleep behavior disorder. *Sleep* **40**, zsw014 (2017). 10.1093/sleep/zsw014 28364450

[166] Joza, S. et al. Prodromal dementia with Lewy bodies in REM sleep behavior disorder: a multicenter study. *Alzheimer’s. Dement.* **20**, 91–102 (2024). 37461299 10.1002/alz.13386 PMC10917000

[167] Wang, J. et al. Visuospatial dysfunction predicts dementia-first phenoconversion in isolated REM sleep behaviour disorder. *J. Neurol. Neurosurg. Psychiatry* **96**, 76–84 (2025). 10.1136/jnnp-2024-333865 38925912

[168] Honeycutt, L. et al. Pareidolias and cognition in isolated REM sleep behavior disorder. *Parkinsonism Relat. Disord.* **75**, 76–79 (2020). 32492550 10.1016/j.parkreldis.2020.05.017

[169] Sadiq, D. et al. Prodromal dementia with Lewy bodies and prodromal Alzheimer’s disease: a comparison of the cognitive and clinical profiles. *J. Alzheimer’s. Dis.* **58**, 463–470 (2017). 28453473 10.3233/JAD-161089

[170] Wyman-Chick, K. A. et al. Prodromal dementia with Lewy bodies: evolution of symptoms and predictors of dementia onset. *J. Geriatr. Psychiatry Neurol.* **35**, 527–534 (2022). 34114509 10.1177/08919887211023586 PMC9150711

[171] Dang-Vu, T. T. et al. Hippocampal perfusion predicts impending neurodegeneration in REM sleep behavior disorder. *Neurology* **79**, 2302–2306 (2012). 23115214 10.1212/WNL.0b013e318278b658 PMC3578380

[172] Iranzo, A. et al. Five-year follow-up of substantia nigra echogenicity in idiopathic REM sleep behavior disorder. *Mov. Disord.* **29**, 1774–1780 (2014). 25384461 10.1002/mds.26055

[173] Liu, Y. et al. Electromyography activity level in rapid eye movement sleep predicts neurodegenerative diseases in idiopathic rapid eye movement sleep behavior disorder: a 5-year longitudinal study. *Sleep. Med.* **56**, 128–134 (2019). 30852128 10.1016/j.sleep.2019.01.018

[174] Miyamoto, T. et al. Striatal dopamine transporter degeneration in right-handed REM sleep behavior disorder patients progresses faster in the left hemisphere. *Parkinsonism Relat. Disord.* **95**, 107–112 (2022). 35093712 10.1016/j.parkreldis.2022.01.015

[175] Rahayel, S. et al. A prodromal brain-clinical pattern of cognition in synucleinopathies. *Ann. Neurol.* **89**, 341–357 (2021). 33217037 10.1002/ana.25962

[176] Laguna, A. et al. Serum metabolic biomarkers for synucleinopathy conversion in isolated REM sleep behavior disorder. *NPJ Parkinsons Dis.* **7**, 40 (2021). 33986284 10.1038/s41531-021-00184-9 PMC8119407

[177] Fernandes, M. et al. Cerebrospinal-fluid biomarkers for predicting phenoconversion in patients with isolated rapid-eye movement sleep behavior disorder. *Sleep* **47**, zsad198 (2024). 10.1093/sleep/zsad198 37542734

[178] Dregan, A., Chowienczyk, P. & Armstrong, D. Patterns of anti-inflammatory drug use and risk of dementia: a matched case–control study. *Eur. J. Neurol.* **22**, 1421–1428 (2015). 26177125 10.1111/ene.12774

[179] Scholz, S. W. et al. Association of cardiovascular disease management drugs with Lewy body dementia: a case–control study. *Brain Commun.* **6**, fcad346 (2023). 38162907 10.1093/braincomms/fcad346 PMC10754316

[180] Numahata, K., Miyamoto, T., Akaiwa, Y. & Miyamoto, M. Brain perfusion single-photon emission computed tomography using an easy Z-score imaging system predicts progression to neurodegenerative dementia in rapid eye movement sleep behavior disorder. *Dement. Geriatr. Cogn. Disord.* **50**, 577–584 (2021). 35100582 10.1159/000521645 PMC9153334

[181] Chiu, S. Y. et al. Sex differences in dementia with Lewy bodies: focused review of available evidence and future directions. *Parkinsonism Relat. Disord.* **107**, 105285 (2023). 36682958 10.1016/j.parkreldis.2023.105285 PMC10024862

[182] Chia, R. et al. Genome sequencing analysis identifies new loci associated with Lewy body dementia and provides insights into its genetic architecture. *Nat. Genet* **53**, 294–303 (2021). 33589841 10.1038/s41588-021-00785-3 PMC7946812

[183] Kimura, T. et al. Whole-genome sequencing to identify rare variants in East Asian patients with dementia with Lewy bodies. *npj Aging* **10**, 52 (2024). 39572598 10.1038/s41514-024-00180-2 PMC11582613

[184] Schwarzerova, J. et al. A perspective on genetic and polygenic risk scores—advances and limitations and overview of associated tools. *Brief Bioinform.* **25**, bbae240 (2024). 10.1093/bib/bbae240 PMC11106636 38770718

[185] Stephan, B. C. M. et al. Population attributable fractions of modifiable risk factors for dementia: a systematic review and meta-analysis. *Lancet Healthy Longev.* **5**, e406–e421 (2024). 38824956 10.1016/S2666-7568(24)00061-8 PMC11139659

[186] Weiss, J. Contribution of socioeconomic, lifestyle, and medical risk factors to disparities in dementia and mortality. *SSM Popul. Health* **16**, 100979 (2021). 34977324 10.1016/j.ssmph.2021.100979 PMC8683757

[187] Liu, C., Murchland, A. R., VanderWeele, T. J. & Blacker, D. Eliminating racial disparities in dementia risk by equalizing education quality: a sensitivity analysis. *Soc. Sci. Med.* **312**, 115347 (2022). 36162365 10.1016/j.socscimed.2022.115347 PMC9990698

[188] Ben-Shlomo, Y. et al. The epidemiology of Parkinson’s disease. *Lancet* **403**, 283–292 (2024). 38245248 10.1016/S0140-6736(23)01419-8 PMC11123577

[189] McKeith, I. G. et al. Research criteria for the diagnosis of prodromal dementia with Lewy bodies. *Neurology* **94**, 743–755 (2020). 32241955 10.1212/WNL.0000000000009323 PMC7274845

[190] Gunawardana, C. W., Matar, E. & Lewis, S. J. G. The clinical phenotype of psychiatric-onset prodromal dementia with Lewy bodies: a scoping review. *J. Neurol.* **271**, 606–617 (2024). 37792074 10.1007/s00415-023-12000-w PMC10769927

[191] Heinzel, S. et al. Update of the MDS research criteria for prodromal Parkinson’s disease. *Mov. Disord.* **34**, 1464–1470 (2019). 31412427 10.1002/mds.27802

[192] Eyting, M. et al. A natural experiment on the effect of herpes zoster vaccination on dementia. *Nature* **641**, 438–446 (2025). 40175543 10.1038/s41586-025-08800-x PMC12058522

[193] Sauerbier, A., Qamar, M. A., Rajah, T. & Chaudhuri, K. R. New concepts in the pathogenesis and presentation of Parkinson’s disease. *Clin. Med.* **16**, 365–370 (2016). 10.7861/clinmedicine.16-4-365 PMC6280220 27481383

[194] Chang, T.-Y., Yang, C.-P., Chen, Y.-H., Lin, C.-H. & Chang, M.-H. Age-stratified risk of dementia in Parkinson’s disease: a nationwide, population-based, retrospective cohort study in Taiwan. *Front Neurol.* **12**, 748096 (2021). 35002917 10.3389/fneur.2021.748096 PMC8740231

[195] Wyman-Chick, K. A. et al. Neuropsychological test performance in mild cognitive impairment with Lewy bodies: a systematic review and meta-analysis. *Alzheimer’s. Dement.* **21**, e14450 (2025). 39791487 10.1002/alz.14450 PMC11848198

[196] Wyman-Chick, K. A., Martin, P. K., Barrett, M. J., Manning, C. A. & Sperling, S. A. Diagnostic accuracy and confidence in the clinical detection of cognitive impairment in early-stage Parkinson disease. *J. Geriatr. Psychiatry Neurol.* **30**, 178–183 (2017). 28351200 10.1177/0891988717701001

[197] Coughlin, D. G., Hurtig, H. I. & Irwin, D. J. Pathological influences on clinical heterogeneity in Lewy body diseases. *Mov. Disord.* **35**, 5–19 (2019). 31660655 10.1002/mds.27867 PMC7233798

[198] Weinshel, S. et al. Appropriateness of applying cerebrospinal fluid biomarker cutoffs from Alzheimer’s disease to Parkinson’s disease. *J. Parkinsons Dis.* **12**, 1155–1167 (2022). 35431261 10.3233/JPD-212989 PMC9934950

[199] Standke, H. G. & Kraus, A. Seed amplification and RT-QuIC assays to investigate protein seed structures and strains. *Cell Tissue Res.* **392**, 323–335 (2023). 35258712 10.1007/s00441-022-03595-z

[200] Wyman-Chick, K. A. et al. Differentiating prodromal dementia with Lewy bodies from prodromal Alzheimer’s disease: a pragmatic review for clinicians. *Neurol. Ther.* **13**, 885–906 (2024). 38720013 10.1007/s40120-024-00620-x PMC11136939

[201] Scott, G. D. et al. Fluid and tissue biomarkers of Lewy body dementia: report of an LBDA symposium. *Front. Neurol.* **12**, 805135 (2021). 35173668 10.3389/fneur.2021.805135 PMC8841880

[202] Gibson, L. L. et al. Risk of dementia in Parkinson’s disease: a systematic review and meta-analysis. *Mov. Disord.* **39**, 1697–1709 (2024). 39036849 10.1002/mds.29918

[203] Hogan, D. B. et al. The prevalence and incidence of dementia with lewy bodies: a systematic review. *Can. J. Neurol. Sci*. **43**, S83–S95 (2016). 10.1017/cjn.2016.2 27307129

[204] Donaghy, P. C. et al. Research diagnostic criteria for mild cognitive impairment with Lewy bodies: a systematic review and meta-analysis. *Alzheimer’s. Dement.* **19**, 3186–3202 (2023). 37096339 10.1002/alz.13105 PMC10695683

[205] Schliep, K. C. et al. How good are medical and death records for identifying dementia?. *Alzheimer’s. Dement.* **18**, 1812–1823 (2022). 34873816 10.1002/alz.12526 PMC9170837

[206] Wilkinson, T. et al. Identifying dementia cases with routinely collected health data: a systematic review. *Alzheimer’s. Dement.* **14**, 1038–1051 (2018). 29621480 10.1016/j.jalz.2018.02.016 PMC6105076

[207] Page, M. J. et al. The PRISMA 2020 statement: an updated guideline for reporting systematic reviews. *BMJ* **372**, n71 (2021). 33782057 10.1136/bmj.n71 PMC8005924

[208] Wells, G. et al. The Newcastle-Ottawa Scale (NOS) for assessing the quality of nonrandomised studies in meta-analyses. https://www.ohri.ca/programs/clinical_epidemiology/oxford.asp (2015).

