# Supplementary material for: Risk factors and predictors for Lewy body dementia: a systematic review
Source: NPJ Dement. 2025 Aug 4;1(1):20. doi: 10.1038/s44400-025-00022-2 (PMC12321581; doi:10.1038/s44400-025-00022-2)
Supplement: Supplementary file 2 — supplementary tables_lbd risk sysrev [file 44400_2025_22_MOESM2_ESM.pdf]

Supplementary Table 1 Non-modifiable factors for Parkinson's disease dementia (PDD) risk and prediction

First author, year	Country	Cohort size	Mean follow-up, years	Sex, % female	Mean age at baseline	Diagnostic criteria	Risk factor	Association (+, /, -)[a]
Stern, 1993 [33]	US	250 PD	2.5	Not reported	68.4	DSM III	Older age (>70), disease severity, depression, levodopa induced confusion or psychosis, hypomimia as presenting sign	+
Breteler, 1995 [42]	Netherlands	4442 PD, 82077 control	8	51.1	66.8	ICD 9 Clinical Modification	Younger age	+
							Sex	/
Jacobs, 1995 [30]	US	122 PD	2.7	41.8	70.5	DSM III Revised	Older age, depression, motor impairment, lower scores on letter fluency, semantic fluency	+
Hughes, 2000 [16]	UK	83 PD, 50 control	4.7	36.1	63.7	DSM III Revised	Older age, motor impairment	+
							Disease duration, age at PD onset	/
Aarsland, 2001 [25]	Norway	130 PD	4	56.0	69.8	DSM III Revised and at least one of: a) MMSE<24; b) At least 3 items on Gottfries, Bråne, and Steen scale ≥ 2 (other than "wakefulness" and "ability to concentrate"); c) UPDRS Intellectual impairment ≥ 2	Older age, MMSE<29, Hoehn and Yahr stage>2	+
Levy, 2002 [19]	US	180 PD, 180 control	3.6	53.9	71.0	DSM III Revised	Older age, disease severity	+
Hobson, 2004 [43]	UK	51 PD	4.4	49.0	74.2	DSM IV	Older age at PD onset, disease severity, hallucination, lower scores on Cambridge Cognitive Examination (CAMCOG) Memory, Language	+

							Education, sex, depression	/
Aarsland, 2007 [26]	Norway, US	487 PD	2.9	51.7	73.5	DSM III Revised	Older age	+
							Age at symptom onset	/
Goris, 2007 [56]	UK	109 PD	3.5	46.3	69.1	DSM IV + MMSE <24	*MAPT* inversion polymorphism	+
Williams-Gray, 2009 [35]	UK	126 PD	5.2	44	69.5	DSM IV	Older age, *MAPT* H1/H1 genotype, impaired semantic fluency, pentagon copying	+
							COMT	/
Kurz, 2009 [54]	Norway	63 PD	9.7	58.7	68.7	DSM III Revised	*APOE*	/
Evans, 2011 [22]	UK	122 PD	5.5	42.6	69.5	DSM IV	Older age, *MAPT* H1/H1 genotype, impaired semantic fluency	+
Rana, 2012 [17]	Canada	310 PD	3.6	43.5	74.8	DSM IV	Older age, disease severity	+
							Sex	/
Nomura, 2013 [31]	Japan	82 PD	1.8	56.1	74.3	MDS PDD	Older age, RBD	+
Winder-Rhodes, 2013 [46]	UK	121 PD	5.7	43.8	67.0	DSM IV + MMSE≤24	*GBA* mutations	+
Alves, 2014 [23]	Norway	104 PD	3.6	31.7	66.6	MDS PDD	Older age, MCI, low CSF amyloid β42 concentrations (ECL <376 pg/mL; ELISA <443 pg/mL)	+
							CSF amyloid β40, amyloid β38, total-tau, phosphorylated-tau	/
Degerman, 2014 [59]	Sweden	136 PD, 30 control	5	41.2	71.4	MDS PDD	Long telomere vs short	+
Kwon, 2014 [37]	South Korea	80 PD	2	62.5	68.1	MDS PDD	Older age (≥62), motor impairment, postural instability gait disorder motor phenotype, vivid dreaming, RBD, hyposmia, depression, abnormal stereopsis	+
Zhu, 2014 [18]	Netherlands	277 PD	4.8	35	58.2	Scales for Outcomes in PD-Cognition (SCOPA-COG)≤22	Older age, depression, excessive daytime sleepiness, higher levodopa dose, Hoehn & Yahr stage	+

Fitts, 2015 [28]	US	132 PD	3.1	31.1	69.4	MDS PDD	Older age, less education, caregiver reported apathy, worse motor impairment, higher levodopa daily dose	+
Cilia, 2016 [47]	Italy	2764 PD	7.2	39.4	57.2	DSM IV	*GBA* mutations; severe *GBA* mutations vs mild	+
Hindle, 2016 [20]	UK	330 PD	4	35.8	66.1	Clinical Dementia Rating≥0.5	Older age, lack of phone use	+
Wu, 2016 [13]	Taiwan	1213 PD	6.3	48.2	62.9	ICD 9 Clinical Modification	Older age, constipation, RBD with constipation, diabetes	+
Anang, 2017 [15]	Canada, Japan	199 PD	4.2	49.6	Not reported	MDS PDD	Older age, male sex, RBD, orthostatic hypotension, MCI	+
							Bilateral disease onset, hallucinations, falls/freezing	/
Backstrom, 2017 [60]	Sweden	133 PD	7.3	39.8	70.5	MDS PDD	*PITX3* C allele carriers	+
Choi, 2017 [24]	South Korea	93 PD	6.7	52.7	65.9	MDS PDD	Older age, low metaiodobenzylguanidine (MIBG) uptake (H/M ratio < 1.35; ^{123}Iodine-MIBG myocardial scintigraphy)	+
Hoogland, 2017 [21]	Canada, Netherlands, New Zealand, US	467 PD	3.3	37.3	68.7	MDS PDD/ MMSE<26 and Functional Independence Measures≥1 item with a score≤5; or MMSE<21	Older age, motor impairment, Level II PD MCI criteria	+
Liu, 2017 [48]	Canada, France, Netherlands, UK, US	3200 PD	3.7	36.2	63.0	MDS PDD	Model with older age at onset, lower MMSE, lower education, motor impairment, male sex, depression, *GBA* mutations	+
Backstrom, 2018 [58]	Sweden	134 PD	7.3	40.3	70.4	MDS PDD	*DRD2* ^{957}T/T genotype	+
Corrado, 2018 [61]	Italy	426 PD	11.3	41.6	73.8	MDS PDD	*SNCA* Rep1 263 carriers	+
Lin, 2018 [57]	Taiwan	409 PD	2	44.3	65.4	MDS PDD	High COMT activity haplotype (G_C_C_G for rs6269, rs4633, rs4818, rs4680)	+

Lunde, 2018 [49]	Norway, Scotland, Sweden	442 PD, 419 control	7	39.6	69.8	MDS PDD/ DSM IV	Deleterious *GBA* mutation or polymorphism carriers	+
Hoogland, 2019 [36]	Canada, Netherlands, New Zealand, US	1045 PD	3.8	36.7	67.7	MDS PDD/ MMSE<26 and Functional Independence Measures≥1 item with a score≤5; or MMSE<21	Older age, male sex, motor impairment, Level I, II PD MCI criteria	+
Phongpreecha, 2020 [40]	US	667 PD	1	36.0	67.0	MDS PDD	*GBA* mutation, male sex, males with *GBA* mutations, older age at PD onset	+
							APOEe4	/
Stoker, 2020 [50]	UK	262 PD	6.8	39.3	69.0	DSM IV	*GBA1* variant carriers	+
Bakeberg, 2021 [38]	Australia	127 PD	3.0	37.0	63.1	ACE-R≤82	Male sex	+
Bakeberg, 2021 [63]	US	368 PD	5	34.5	61.8	MDS PDD	*TOMM40* '523' allele length, when considered with *APOE* genotype	+
Galtier, 2021 [27]	Spain	42 PD, 19 control	7.5	42.9	59.2	MDS PDD	Older age (≥65), MCI, combination of Judgment of Line Orientation errors QO2 (oblique line confused with another different oblique line from the same quadrant separated by two or three spacings of 18º) & IQO (oblique line from one quadrant is displaced to the other quadrant)	+
							Disease duration, age at PD onset, motor impairment, side of disease onset	/
Horne, 2021 [29]	New Zealand	202 PD	4	32.2	68.7	MDS PDD	Older age, worse global cognition (derived from tests across attention/working memory, executive, visuospatial, memory, language domains)	+
Liu, 2021 [52]	Canada, Estonia, France, Germany, Netherlands,	8605 PD	6.7	37.3	64.5	MDS PDD/ UPDRS Intellectual impairment=4/ Cognitive impairment leading to functional impairment	*APOE*, *GBA*, *RIMS2* (rs182987047), *TMEM108* (rs138073281), *WWOX* (rs8050111)	+

	Norway, UK, US							
Counsell, 2022 [45]	Scotland	201 PD, 260 control	7.5	39.0	72.6	MDS PDD	Older age at PD onset, subjective cognitive symptoms, RBD, motor impairment, lower MMSE, *ApoEe4* allele	+
							Sex	/
Fang, 2022 [62]	US	382 PD, 180 control	5.5	34.0	61.8	MDS PDD	*AQP4* A allele carriers (rs162009)	-
Jeong, 2022 [41]	South Korea	571 PD	6.0	51.1	64.0	MDS PDD	Male sex, increased total white matter hyperintensities (MRI), reduced dopamine transporter availability in anterior putamen (^{18}F-FP-CIT PET)	+
Myers, 2022 [55]	US	152 PD, 52 control	4.1	38.8	66.7	Clinical Dementia Rating≥1	CSF, PET amyloid β positivity (^{11}C-Pittsburgh Compound-B PET), *APOEe4*	+
Szwedo, 2022 [51]	Norway, Sweden, UK	1002 PD	5.4	39.0	68.9	MDS PDD	*APOEe4* genotype, *GBA* mutation carriers, *APOEe4* & *GBA* interaction	+
							MAPT, SNCA (rs356219)	/
Umeh, 2022 [44]	US	130 PD	5.5	36.2	79.5	DSM IV	*APOEe4* genotype	+
							Sex, sex & *APOEe4* interaction	/
Vijiaratnam, 2022 [53]	UK	258 PD	3.7	36.3	68.4	MDS PDD	Older age, male sex, *APOEe4* genotype, motor impairment, higher serum neurofilament light chain	+
							GBA	/
Bohn, 2023 [14]	Canada	48 PD	3	56	71.6	MDS PDD	Model with older age (>70), male sex, falls and/or freezing of gait, bilateral disease, RBD, orthostatic hypotension, MCI, visual hallucinations	+
Fink, 2023 [39]	Germany	2195 PD	3.9	55.2	77.6	ICD 10	Older age at PD onset, male sex, disease severity	+
							Sex after accounting for sex-specific survival patterns	/

McFall, 2023 [34]	Canada	48 PD	3.0	43.8	71.5	DSM IV	Model with older age, worse gait, activities of daily living, Trail Making Test A, B, Choice reaction time, word recall, larger third ventricle volume (MRI), up-regulated metabolites (Hydroxy-isoleucine, His-Asn-Asp-Ser, Alanyl-alanine, Putrescine [-2H], 3,4-Dihydroxyphenylacetone) and creatinine (blood)	+
Park, 2023 [32]	South Korea	262 PD	7.1	48.9	68.2	MDS PDD	Model with older age, disease duration, lower composite scores of visuospatial/visual memory, verbal memory, frontal/executive function, increased caudate texture heterogeneity (MRI)	+
Tunold, 2024 [64]	US	1151 PD	2.4	35.5	63.7	Montreal Cognitive Assessment≤21	Lysosomal pathway-specific polygenic risk score in people with low likelihood for Alzheimer's disease co-pathology	+

[a]Association with PDD marked with - for reduced risk, + for increased risk, / for no significant association.
CSF: cerebrospinal fluid, DSM: The Diagnostic and Statistical Manual of Mental Disorders, ICD: International Classification of Diseases, MCI: mild cognitive impairment, MDS: International Parkinson and Movement Disorder Society, MMSE: Mini Mental State Exam, MRI: magnetic resonance imaging, PET: positron emission tomography, PD: Parkinson's disease, PDD: Parkinson's disease dementia, RBD: rapid eye movement sleep behavior disorder, UPDRS: Unified Parkinson's Disease Rating Scale

Supplementary Table 2 Modifiable factors for Lewy body dementia (LBD) risk and prediction

Outcome	First author, year	Country	Cohort size	Mean follow-up, years	Sex, % female	Mean age at baseline	Diagnostic criteria	Risk factor	Association (+, /, -)[a]
PDD	Ebmeier, 1990 [67]	Scotland	127 PD	3.6	55.9	73.6	DSM III Revised	Smoking, upper limb bradykinesia, speech changes, Hoehn and Yahr stage, mental status questionnaire	+
	Stern, 1993 [33]	US	250 PD	2.5	Not reported	68.4	DSM III	Older age (>70), disease severity, depression, levodopa induced confusion or psychosis, hypomimia as presenting sign	+
	Jacobs, 1995 [30]	US	122 PD	2.7	41.8	70.5	DSM III Revised	Older age, depression, motor impairment, lower scores on letter fluency, semantic fluency	+
	Levy, 2002 [68]	US	180 PD	3.6	55.6	71.0	DSM III Revised	Current smoker vs non-smoker; ever-smoked vs non-smoker	+
								Head injury, hypertension, diabetes	/
	Aarsland, 2003 [86]	Norway	224 PD	8	52	73.4	DSM III Revised and at least one of: a) MMSE<age- and education-based lower quartile; b) At least 3 items on Gottfries, Bråne, and Steen scale ≥ 2 (other than "wakefulness" and "ability to concentrate"); c)	Hallucinations, akinetic phenotype	+
								Symmetrical PD	/

							UPDRS Intellectual impairment ≥ 2		
	Hobson, 2004 [43]	UK	51 PD	4.4	49.0	74.2	DSM IV	Older age at PD onset, disease severity, hallucination, lower scores on Cambridge Cognitive Examination (CAMCOG) Memory, Language	+
								Education, sex, depression	/
	Haugarvoll, 2005 [93]	Norway	130 PD	4	50.9	69.8	DSM III Revised and at least one of: a) MMSE<age- and education-based lower quartile; b) At least 3 items on Gottfries, Bråne, and Steen scale ≥ 2 (other than “wakefulness” and “ability to concentrate”); c) UPDRS Intellectual impairment ≥ 2	Cerebrovascular risk factors including coronary heart disease, atrial fibrillation, heart failure, stroke/transient ischemic attack, hypertension, diabetes, smoking	/
	Ramirez-Ruiz, 2007 [90]	Spain	40 PD, 18 control	1	57.5	74.1	DSM IV Text Revision	Visual hallucinations	+
	Baba, 2012 [80]	Japan	44 PD	3	47.7	65.3	MDS PDD	Hyposmia, higher number of erroneously identified objects not in the overlapping-figure identification test	+
	Postuma, 2012 [72]	Canada	42 PD	4.0	19.1	69.4	MDS PDD	RBD	+

	Bugalho, 2013 [87]	Portugal	61 PD	2	54.1	71.9	DSM IV Text Revision	Worse speech, rigidity, gait/posture symptoms, non-tremor-dominant motor phenotype, hallucinations	+
	Nomura, 2013 [31]	Japan	82 PD	1.8	56.1	74.3	MDS PDD	Older age, RBD	+
	Anang, 2014 [71]	Canada	80 PD	4.4	36.3	66.2	MDS PDD	Orthostatic hypotension, RBD, color vision, MCI, gait dysfunction	+
	Kwon, 2014 [37]	South Korea	80 PD	2	62.5	68.1	MDS PDD	Older age (≥62), motor impairment, postural instability gait disorder motor phenotype, vivid dreaming, RBD, hyposmia, depression, abnormal stereopsis	+
	Sanyal, 2014 [91]	India	250 PD, 280 control	7	35.2	57.9	DSM III Revised	Hallucinations, akinetic vs tremor-dominant motor phenotype, asymmetrical vs symmetrical disease onset	+
	Zhu, 2014 [18]	Netherlands	277 PD	4.8	35	58.2	Scales for Outcomes in PD-Cognition (SCOPA-COG)≤22	Older age, depression, excessive daytime sleepiness, higher levodopa dose, Hoehn & Yahr stage	+
	Fitts, 2015 [28]	US	132 PD	3.1	31.1	69.4	MDS PDD	Older age, less education, caregiver reported apathy, worse motor impairment, higher levodopa daily dose	+
	Hindle, 2016 [20]	UK	330 PD	4	35.8	66.1	Clinical Dementia Rating≥0.5	Older age, lack of phone use	+
	Wu, 2016 [13]	Taiwan	1213 PD	6.3	48.2	62.9	ICD 9 Clinical Modification	Older age, constipation, RBD with constipation, diabetes	+
	Anang, 2017 [15]	Canada, Japan	199 PD	4.2	49.6	Not reported	MDS PDD	Older age, male sex, RBD, orthostatic hypotension, MCI	+

								Bilateral disease onset, hallucinations, falls/freezing	/
	Domellof, 2017 [82]	Sweden	125 PD	5	40	70.6	MDS PDD	Hyposmia	+
	Lee, 2017 [65]	South Korea	1193 PD	3.9	66.3	64.3	DSM IV	Older age at PD onset, education	+
	Liu, 2017 [48]	Canada, France, Netherlands, UK, US	3200 PD	3.7	36.2	63.0	MDS PDD	Model with older age at onset, lower MMSE, lower education, motor impairment, male sex, depression, *GBA* mutations	+
	Peng, 2018 [85]	China	787 PD	5	43.1	67.1	MDS PDD	Treatment for metabolic syndrome vs no treatment; treated for all main components of metabolic syndrome vs treated for some components; treatment for hypertension, high fasting plasma glucose and hypertriglyceridemia vs no treatment	-
	Hong, 2019 [99]	Taiwan	21934 PD	5.4	30.8	69.4	ICD 9 Clinical Modification/ ICD 10 + at least one global cognitive screening (MMSE, Clinical Dementia Rating, Cognitive Abilities Screening Instrument)	Exposure to antiparkinsonism anticholinergics for ≥6 months	+
	Sheu, 2019 [98]	Taiwan	1232 PD	4	44.2	Not reported	ICD 9 Clinical Modification	Type of the anticholinergics, classified by the burden of the anticholinergic effect	/
								Cumulative dose effect of anticholinergics	+

	Yoo, 2019 [100]	South Korea	119 PD treated with levodopa>5 years	7.3	52.1	67.4	MDS PDD	Levodopa induced dyskinesia	+
	Yoo, 2019 [95]	South Korea	21 underweight/normal PD, 22 overweight PD, 27 obese PD	7.9	44.3	66.3	MDS PDD	Body mass index (BMI)≥23 kg/m^2 (overweight + obese) vs BMI<23 kg/m^2 (normal/underweight)	-
	Campos-Sousa, 2020 [83]	Brazil	63 PD	7	100	64.1	MoCA<22	Detrusor overactivity	+
	Gryc, 2020 [88]	US	444 PD	4.5	34.2	67.9	MDS PDD	Caregiver reported hallucinations	+
	Tajiri, 2020 [74]	Japan	49 PD MCI	1.5	51.0	70.3	MDS PDD	Lower MMSE, lightheadedness, impulse control disorders	+
	Bejr-kasem, 2021 [89]	US	131 PD	5	35.1	60.8	MDS PDD	Major psychosis with well-formed hallucinations or delusions	+
	Jurcau, 2021 [70]	Romania	89 PD	3	40.4	66.1	DSM IV	Autonomic dysfunction, insomnia	+
	Jeong, 2021 [97]	South Korea	494 PD	5.4	52.2	64.7	MDS PDD	Statins, independent of total cholesterol	+
	Leta, 2021 [69]	Argentina, Austria, Brazil, Canada, Ecuador, Germany, Greece, India, Israel, Italy, Japan, Mexico, Netherlands, Nigeria, Romania, Sweden, Spain, UK, US, Venezuela	619 PD	4.9	35.9	64.0	MDS PDD	Constipation	+

	Liang, 2021 [77]	Taiwan	1694 PD	3	52.1	78.3	ICD 9 Clinical Modification	Chinese herbal medicine, statins	-
	Liang, 2021 [77]	Taiwan	1694 PD	3	52.1	78.3	ICD 9 Clinical Modification	Chronic kidney disease, chronic obstructive pulmonary disease, stroke.	+
	Onofrj, 2021 [78]	Italy	639 PD with no genetic mutations	12	36.0	69.5	MMSE<18	Bipolar spectrum disorder	+
	Backstrom, 2022 [81]	Sweden	143 PD	10	40.6	71.2	MDS PDD	Model including MCI, worse motor impairment, hyposmia, lower CSF amyloid β42	+
	Borda, 2022 [66]	Norway	192 PD, 171 control	3	39.1	68.1	MDS PDD	Frailty	+
	Counsell, 2022 [45]	Scotland	201 PD, 260 control	7.5	39.0	72.6	MDS PDD	Older age at PD onset, subjective cognitive symptoms, RBD, motor impairment, lower MMSE, *ApoEe4* allele	+
	Counsell, 2022 [45]	Scotland	201 PD, 260 control	7.5	39.0	72.6	MDS PDD	Sex	/
	Longardner, 2022 [75]	Italy	50 PD	5	40	64.3	MoCA<21	Cardiovascular dysautonomia	+
	Bohn, 2023 [14]	Canada	48 PD	3	56	71.6	MDS PDD	Model with older age (>70), male sex, falls and/or freezing of gait, bilateral disease, RBD, orthostatic hypotension, MCI, visual hallucinations	+
	Jung, 2023 [96]	South Korea	150 PD	5.2	40.7	70.8	MDS PDD	Dihydropyridine calcium channel blocker	-
	Jung, 2023 [96]	South Korea	150 PD	5.2	40.7	70.8	MDS PDD	Angiotensin receptor blocker, beta-blocker, alpha-blocker, diuretics	/
	Kang, 2023 [76]	South Korea	79622 PD	12.5	60.4	70.0	ICD 10	Older age at PD diagnosis, longer disease duration, hypertension, diabetes, dyslipidemia, depression	+

	Lee, 2023 [92]	South Korea	338 PD MCI	3.8	52.4	69.9	MDS PDD	Caregiver reported mood change, hyperactivity, psychosis	+
	Lee, 2023 [155]	South Korea	124 PD	5.3	55.6	74.1	MDS PDD	Higher levodopa equivalent daily dose, motor impairment, lower MMSE, lower serum zinc	+
	Qu, 2023 [94]	US	508 PD, 168 control	10	37.6	62.1	MoCA<22	Estimated glomerular filtration rate, renal function insufficiency	/
	Gerakios, 2024 [79]	UK	121 PD, 199 control	1	38.8	76.8	DSM V	Delirium	+
	Kang, 2024 [84]	South Korea	9264 PD	9.5	50.2	67.9	ICD 10	Higher visit-to-visit glucose variability, even without diabetes	+
	Zhong, 2024 [73]	US	413 PD, 196 control	5	34.9	61.5	MoCA<22	RBD; multiple sleep disturbances vs none	+
DLB	Iranzo, 2013 [161]	Spain	44 RBD, 20 control	10.5	11.4	74	McKeith 2005	RBD	+
	Jung, 2017 [162]	US	15 RBD	6.4	20	81.1	McKeith 2005	RBD	+
	Wang, 2020 [164]	Hong Kong	133 RBD	2.6	22.6	66.5	McKeith 2005	Residual injurious symptoms after clonazepam/melatonin	+
	Shim, 2022 [158]	South Korea	92095 with herpes simplex virus, 97323 varicella zoster virus, 183779 control	10	56.0	62.5	ICD 10	Herpes simplex virus	+
								Varicella zoster virus	/
	Zolfaghari, 2022 [163]	Canada	183 RBD	4.4	24.6	66.4	McKeith 2017	Cardiovascular disorders, hypertension, hypercholesterolemia, diabetes	/
	Golimstok, 2024 [159]	Argentina	161 with attention deficit hyperactivity disorder, 109 without	12.1	54	58.2	McKeith 2017	Attention deficit hyperactivity disorder	+
	Hart, 2024 [160]	US	126313 taking terazosin, doxazosin, or alfuzosin, 437045 taking tamsulosin,	2.2	0	62.6	ICD 9 Clinical Modification/ ICD 10	Terazosin/doxazosin/alfuzosin vs tamsulosin/5α-reductase inhibitor	-
								Tamsulosin vs 5α-reductase inhibitor	Similar

			80158 taking 5α-reductase inhibitor						
LBD	Dregan, 2015 [178]	UK	1694 LBD, 31083 Alzheimer's disease, 23465 vascular dementia, 350661 control	9.9[b]	42	70	Read codes	Prescription of NSAIDs or glucocorticoids >10 years prior to dementia	+
								History of any NSAID glucocorticoid prescription	/
	Scholz, 2023 [179]	US	148170 LBD, 1253043 control	3[b]	49.1	79.5	ICD 9 Clinical Modification	Medications to treat cardiovascular diseases (anti-hypertensives, cholesterol-lowering agents, anti-diabetics)	-

[a]Association with PDD, DLB, or LBD marked with - for reduced risk, + for increased risk, / for no significant association. [b]Retrospective
CSF: cerebrospinal fluid, DLB: dementia with Lewy bodies, DSM: The Diagnostic and Statistical Manual of Mental Disorders, ICD: International Classification of Diseases, LBD: Lewy body dementia, MCI: mild cognitive impairment, MDS: International Parkinson and Movement Disorder Society, MMSE: Mini Mental State Exam, MoCA: Montreal Cognitive Assessment, PD: Parkinson's disease, PDD: Parkinson's disease dementia, RBD: rapid eye movement sleep behavior disorder, UPDRS: Unified Parkinson's Disease Rating Scale

Supplementary Table 3 Clinical factors for Lewy body dementia (LBD) risk and prediction

Outcome	First author, year	Country	Cohort size	Mean follow-up, years	Sex, % female	Mean age at baseline	Diagnostic criteria	Risk factor	Association (+, /, -)[a]
PDD	Ebmeier, 1990 [67]	Scotland	127 PD	3.6	55.9	73.6	DSM III Revised	Smoking, upper limb bradykinesia, speech changes, Hoehn and Yahr stage, mental status questionnaire	+
	Stern, 1993 [33]	US	250 PD	2.5	Not reported	68.4	DSM III	Older age (>70), disease severity, depression, levodopa induced confusion or psychosis, hypomimia as presenting sign	+
	Jacobs, 1995 [30]	US	122 PD	2.7	41.8	70.5	DSM III Revised	Older age, depression, motor impairment, impaired letter fluency, semantic fluency	+
	Marder, 1995 [128]	US	140 PD, 572 control	2	52.9	71.1	DSM III Revised	Motor impairment	+
								Age at motor symptom onset	/
	Mahieux, 1998 [118]	France	81 PD	3.5	43.2	66.9	DSM III Revised	Age at PD onset (>60), WAIS-R Picture Completion (<10), Stroop Color (<21), letter fluency (<9)	+
	Hughes, 2000 [16]	UK	83 PD, 50 control	4.7	36.1	63.7	DSM III Revised	Older age, motor impairment	+
								Disease duration, age at PD onset	/
	Aarsland, 2001 [25]	Norway	130 PD	4	56.0	69.8	DSM III Revised and at least one of: a) MMSE<24; b) At least 3 items on Gottfries, Bråne, and Steen scale ≥ 2 (other than “wakefulness” and “ability to concentrate”); c)	Older age, MMSE<29, Hoehn and Yahr stage>2	+

							UPDRS Intellectual impairment ≥ 2		
	Levy, 2002 [19]	US	180 PD, 180 control	3.6	53.9	71.0	DSM III Revised	Older age, disease severity	+
	Aarsland, 2003 [86]	Norway	224 PD	8	52	73.4	DSM III Revised and at least one of: a) MMSE<age- and education-based lower quartile; b) At least 3 items on Gottfries, Bråne, and Steen scale ≥ 2 (other than “wakefulness” and “ability to concentrate”); c) UPDRS Intellectual impairment ≥ 2	Hallucinations, akinetic motor phenotype	+
								Symmetrical PD	/
	Hobson, 2004 [43]	UK	51 PD	4.4	49.0	74.2	DSM IV	Older age at PD onset, disease severity, hallucination, lower scores on Cambridge Cognitive Examination (CAMCOG) Memory, Language	+
								Education, sex, depression	/
	Alves, 2006 [129]	Norway	171 PD	8	50.9	71.3	DSM III Revised	Transition from tremor dominant or indeterminate to postural instability gait disorder motor phenotype	+
	Burn, 2006 [130]	UK	40 PD, 41 control	2	35.8	75.3	DSM IV	Postural instability gait disorder motor phenotype	+
	Janvin, 2006 [111]	Norway	72 PD	4	55.6	71.0	DSM III Revised	Single domain non-amnestic MCI, multi domain slightly impaired MCI	+
								Amnestic MCI	/
	Aarsland, 2007 [26]	Norway, US	487 PD	2.9	51.7	73.5	DSM III Revised	Older age	+
								Age at symptom onset	/

	Gago, 2009 [132]	Portugal	24 PD, 20 control	6	37.5	63.7	DSM IV	Speech impairment	+
	Williams-Gray, 2009 [35]	UK	126 PD	5.2	44	69.5	DSM IV	Older age, *MAPT* H1/H1, impaired semantic fluency, pentagon copying	+
								COMT	/
	Evans, 2011 [22]	UK	122 PD	5.5	42.6	69.5	DSM IV	Older age, *MAPT* H1/H1, impaired semantic fluency	+
	Baba, 2012 [80]	Japan	44 PD	3	47.7	65.3	MDS PDD	Hyposmia, higher number of erroneously identified objects that are not in the overlapping-figure identification test	+
	Rana, 2012 [17]	Canada	310 PD	3.6	43.5	74.8	DSM IV	Older age, disease severity	+
								Sex	/
	Bugalho, 2013 [87]	Portugal	61 PD	2	54.1	71.9	DSM IV Revised	Worse speech, rigidity, gait/posture symptoms, non-tremor motor phenotype, hallucinations	+
	Compta, 2013 [122]	Spain	27 PD	1.5	30.0	69.0	MDS PDD	Low CSF amyloid β, impaired verbal learning, semantic fluency, visuoperception, frontal and anterior cingulate cortical thinning (MRI)	+
	Pedersen, 2013 [106]	Norway	182 PD	3	40.1	67.5	MDS PDD	MCI, regardless of persistence or reversion	+
	Anang, 2014 [71]	Canada	80 PD	4.4	36.3	66.2	MDS PDD	Orthostatic hypotension, RBD, color vision, MCI, gait dysfunction	+
	Dubbelink, 2014 [116]	Netherlands	63 PD	7.0	38.1	62.4	MDS PDD	Combination of impaired fronto-executive task (spatial span) and low beta power (magnetoencephalography)	+
	Gasca-Salas, 2014 [110]	Spain	49 PD, 62 control	2.6	40.5	69.1	MDS PDD	Multi domain MCI, impaired Stroop Word, pentagon copy, Raven Progressive Matrices	+
	Kwon, 2014 [37]	South Korea	80 PD	2	62.5	68.1	MDS PDD	Older age (≥62), motor impairment, postural instability gait disorder	+

								phenotype, vivid dreaming, RBD, hyposmia, depression, abnormal stereopsis	
	Lee, 2014 [117]	South Korea	51 PD MCI	2.6	33.3	71.4	MDS PDD	Impaired Stroop Word, Color Word, semantic fluency	+
	Sanyal, 2014 [91]	India	250 PD, 280 control	7	35.2	57.9	DSM III Revised	Hallucinations, akinetic vs tremor dominant phenotype, asymmetrical vs symmetrical disease onset	+
	Zhu, 2014 [18]	Netherlands	277 PD	4.8	35	58.2	Scales for Outcomes in PD-Cognition (SCOPA-COG)≤22	Older age, depression, excessive daytime sleepiness, higher levodopa dose, Hoehn & Yahr stage	+
	Domellof, 2015 [28,104]	Sweden	134 PD	5	30.6	71.3	MDS PDD	MCI	+
	Fitts, 2015 [28]	US	132 PD	3.1	31.1	69.4	MDS PDD	Older age, less education, caregiver reported apathy, worse motor impairment, higher levodopa daily dose	+
	Galtier, 2016 [125]	Spain	43 PD, 20 control	6 to 8	44.2	59.2	MDS PDD	Memory decline, higher Hoehn and Yahr stage	+
	Kurlan, 2016 [113]	US	491 PD	5.5	34.0	59.6 (9.4)	MMSE<24/ MoCA<26	Disease stage, lower MMSE, Schwab & England Activities of Daily Living	+
	Lee, 2016 [131]	South Korea	96 PD	6.24	71.9	70.6	DSM IV	Postural instability	+
	Anang, 2017 [15]	Canada, Japan	199 PD	4.2	49.6	Not reported	MDS PDD	Older age, male sex, RBD, orthostatic hypotension, MCI	+
								Bilateral disease onset, hallucinations, falls/freezing	/
	Hoogland, 2017 [21]	Canada, Netherlands, New Zealand, US	467 PD	3.3	37.3	68.7	MDS PDD/ MMSE<26 and Functional Independence Measures≥1 item with	Older age, motor impairment, Level II PD MCI criteria	+

							a score≤5; or MMSE<21		
	Lee, 2017 [65]	South Korea	1193 PD	3.9	66.3	64.3	DSM IV	Older age at PD onset, education	+
	Liu, 2017 [48]	Canada, France, Netherlands, UK, US	3200 PD	3.7	36.2	63.0	MDS PDD	Model with older age at onset, lower MMSE, lower education, motor impairment, male sex, depression, *GBA*	+
	Modreanu, 2017 [133]	Spain	37 PD	1.5	38	64.0	MDS PDD	Non-motor predominance (≥3 non-motor symptoms with ≥2 being moderate-severe and ≥1 being present from onset), lower CSF amyloid β	+
	Pedersen, 2017 [105]	Norway	164 PD	5	39	67.3	MDS PDD	MCI, regardless of persistence or reversion	+
	Ye, 2017 [115]	South Korea	216 PD	2.7	51.4	68.9	MDS PDD	Impaired naming, semantic fluency, frontal/executive function, immediate verbal memory, visuospatial function tests	+
	Cholerton, 2018 [123]	US	332 PD MCI	3.2	26.8	68.0	MDS PDD	Impaired processing speed, working memory	+
	Chung, 2019 [112]	South Korea	186 PD MCI	1 to 4	45.2	69.5	Not reported	Amnestic MCI vs non-amnestic MCI	+
	Galtier, 2019 [126]	Spain	43 PD, 20 control	7.5	44.2	59.2	MDS PDD	Deficits in memory, language	+
	Hoogland, 2019 [36]	Canada, Netherlands, New Zealand, US	1045 PD	3.8	36.7	67.7	MDS PDD/ MMSE<26 and Functional Independence Measures≥1 item with a score≤5; or MMSE<21	Older age, male sex, motor impairment, Level I, II PD MCI criteria	+
	Jalakas, 2019 [114]	Sweden	175 PD, 51 control	5.5	37.1	65	MDS PDD	Impaired clock drawing, A Quick Test of cognitive speed	+

	Massa, 2019 [124]	Italy	24 PD, 18 control	5.0	29.2	74.8	Not reported	Impaired Corsi span, semantic fluency, temporal lobe hypometabolism (FDG PET), reduced putamen/caudate (DAT SPECT)	+
	Nicoletti, 2019 [107]	Italy	139 PD	2	37.4	65.7	MDS PDD	MCI; multi domain MCI vs single domain; executive then attention deficits	+
	Campbell, 2020 [103]	US	162 PD	4.8	38.3	66.1	MDS PDD	Impaired cognition and moderate motor deficits subtype vs motor only (characterized by primary motor deficits), psychiatric & motor (characterized by prominent psychiatric symptoms and moderate motor deficits)	+
	Chung, 2020 [119]	South Korea	350 PD	5.6	52.3	67.9	MDS PDD	Deficits in frontal/executive, attention, working memory, language, verbal memory, visual memory, visuospatial	+
	De Roy, 2020 [109]	Canada	80 PD	4.3	31	66.3	MDS PDD	Multi domain MCI, deficits in Block Design, Stroop Color Word, Rey Auditory Verbal Learning Test, semantic fluency	+
	Phongpreecha, 2020 [40]	US	667 PD	1	36.0	67.0	MDS PDD	*GBA* mutation, male sex, males with *GBA* mutations, older age at PD onset	+
	Tajiri, 2020 [74]	Japan	49 PD MCI	1.5	51.0	70.3	MDS PDD	Lower MMSE, lightheadedness, impulse control disorders	+
	Galtier, 2021 [27]	Spain	42 PD, 19 control	7.5	42.9	59.2	MDS PDD	Older age (≥65), MCI, combination of Judgment of Line Orientation errors QO2 (oblique line confused with another different oblique line from the same quadrant separated by two or three spacings of 18º) & IQO (oblique line from one quadrant is displaced to the other quadrant)	+

								Disease duration, age at PD onset, motor impairment, side of disease onset	/
	Horne, 2021 [29]	New Zealand	202 PD	4	32.2	68.7	MDS PDD	Older age, worse global cognition (derived from tests across attention/working memory, executive, visuospatial, memory, language domains)	+
	Nicoletti, 2021 [108]	Italy	139 PD	2.0	37.3	65.7	MDS PDD	Male sex, MCI, higher Wahlund score for presence of white matter lesions (MRI)	+
	Backstrom, 2022 [81]	Sweden	143 PD	10	40.6	71.2	MDS PDD	Model including MCI, worse motor impairment, hyposmia, lower CSF amyloid β42	+
	Becker, 2022 [101]	Germany	164 PD	3.8	37.3	66.2	MDS PDD	Functional Assessment Questionnaire>1 (Cognitive instrumental activities of daily living scale)	+
	Boel, 2022 [102]	US, Canada, Netherlands, New Zealand	467 PD	3.3	37.3	68.7	MDS PDD/ MMSE<26 and Functional Independence Measures≥1 item with a score≤5; or MMSE<21	Level I PD MCI with MMSE, MoCA	+
	Chung, 2022 [127]	South Korea	163 PD	4.2	54	70.4	MDS PDD	Higher motor reserve based on higher UPDRS-III and dopamine transporter availability in the posterior putamen (^{18}F-FP-CIT PET)	-
	Counsell, 2022 [45]	Scotland	201 PD, 260 control	7.5	39.0	72.6	MDS PDD	Older age at PD onset, subjective cognitive symptoms, RBD, motor impairment, lower MMSE, *ApoEe4*	+
								Sex	/
	Vijiaratnam, 2022 [53]	UK	258 PD	3.7	36.3	68.4	MDS PDD	Older age, male sex, *APOEe4*, motor impairment, higher serum neurofilament light chain	+

								GBA	/
	Bohn, 2023 [14]	Canada	48 PD	3	56	71.6	MDS PDD	Model with older age (>70), male sex, falls and/or freezing of gait, bilateral disease, RBD, orthostatic hypotension, MCI, visual hallucinations	+
	Fink, 2023 [39]	Germany	2195 PD	3.9	55.2	77.6	ICD 10	Older age at PD onset, male sex, disease severity	+
								Sex after accounting for sex-specific survival patterns	/
	Galtier, 2023 [120]	Spain	46 PD, 20 control	7.5	47.8	59.3	MDS PDD	Impaired action naming, action generation	+
	Kang, 2023 [76]	South Korea	79622 PD	12.5	60.4	70.0	ICD 10	Older age at PD diagnosis, longer disease duration, hypertension, diabetes, dyslipidemia, depression	+
	Lee, 2023 [155]	South Korea	124 PD	5.3	55.6	74.1	MDS PDD	Higher levodopa equivalent daily dose, higher UPDRS III, lower MMSE, lower serum zinc	+
	McFall, 2023 [34]	Canada	48 PD	3.0	43.8	71.5	DSM IV	Model with older age, worse gait, activities of daily living, Trail Making Test A, B, Choice reaction time, word recall, larger third ventricle volume (MRI), up-regulated metabolites (Hydroxy-isoleucine, His-Asn-Asp-Ser, Alanyl-alanine, Putrescine [-2H], 3,4-Dihydroxyphenylacetone) and creatinine (blood)	+
	Park, 2023 [32]	South Korea	262 PD	7.1	48.9	68.2	MDS PDD	Model with older age, disease duration, lower composite scores of visuospatial/visual memory, verbal memory, frontal/executive function, increased caudate texture heterogeneity (MRI)	+

	Summers, 2024 [121]	US	312 PD	5	34.0	60.8	MDS PDD	Posterior cortical vs indeterminate cognitive profile	+
								Posterior cortical vs frontostriatal cognitive profile; tremor-dominant/indeterminate motor vs postural instability gait disorder motor phenotype	/
DLB	Marchand, 2017 [165]	Canada	92 RBD, 30 control	3.6	26	67.4	McKeith 2005 DLB	Stroop Color Word (III–II, time), Trail Making Test B, letter fluency, semantic fluency, Rey Auditory Verbal Learning Test	+
	Sadiq, 2017 [169]	UK	292 MCI	4.7	49.0	73.6	McKeith 2005 DLB	Parkinsonism, fluctuating cognition, RBD, visuospatial deficit, impaired letter fluency	+
	Honeycutt, 2020 [168]	Canada	100 RBD	1.6	18	67.7	Not reported	False noise errors on pareidolia test	+
	Rahayel, 2021 [175]	Australia, Canada	76 RBD	3.4	17	66.7	McKeith 2017, DSM V	Model with clinical score (MCI, akinetic-rigid motor phenotype) and brain deformation signature (atrophy in the basal ganglia, thalamus, amygdala, frontotemporal grey and white matter, subarachnoid/ventricular expansion; MRI)	+
	Wyman-Chick, 2022 [170]	US	116 DLB, 348 control	2.0*	17.2	75.6	Available McKeith criteria at the time of assessment	≥ 1 DLB core clinical feature (cognitive fluctuations, visual hallucinations, RBD, parkinsonism) in combination with apathy, depression, or anxiety; ≥ 2 DLB core clinical features	+
	Joza, 2024 [166]	Australia, Austria, Canada, China, Czech Republic,	754 RBD	3.3	20.3	67.4	DSM V	MCI, attention decline (Trail Making Test A, Stroop interference), executive decline (Trail Making Test B, B-A, semantic fluency), memory decline (Word list immediate recall),	+

		France, Italy, Germany, South Korea, UK, US						impairment on digit span backward, letter fluency, figure copy, naming	
	Wang, 2024 [167]	Hong Kong	175 RBD, 98 control	5.1	25.7	67.7	McKeith 2005	Lower Rey–Osterrieth complex figure (ROCF)-copy, lower composite score of Color Trails Test and ROCF, lower MoCA	+

[a]Association with PDD, DLB, or LBD marked with - for reduced risk, + for increased risk, / for no significant association.

CSF: cerebrospinal fluid, DLB: dementia with Lewy bodies, DAT SPECT: dopamine transporter single photon emission computed tomography, DSM: The Diagnostic and Statistical Manual of Mental Disorders, FDG PET: fludeoxyglucose positron emission tomography, ICD: International Classification of Diseases, MCI: mild cognitive impairment, MDS: International Parkinson and Movement Disorder Society, MMSE: Mini Mental State Exam, MoCA: Montreal Cognitive Assessment, PD: Parkinson's disease, PDD: Parkinson's disease dementia, PET: positron emission tomography, RBD: rapid eye movement sleep behavior disorder, UPDRS: Unified Parkinson's Disease Rating Scale

Supplementary Table 4 Imaging and fluid biomarkers for Lewy body dementia (LBD) risk and prediction

Outcome	First author, year	Country	Cohort size	Mean follow-up, years	Sex, % female	Mean age at baseline	Diagnostic criteria	Risk factor	Association (+, /, -)[a]
PDD	De Weerd, 1990 [138]	Netherlands	62 PD	3	72.6	70.7	DSM III + MMSE<23	Focal abnormalities, higher grand total of EEG	+
	Chen-Plotkin, 2011 [156]	US	54 PD	1.8	18.5	71.0	Age-adjusted Mattis Dementia Rating Scale-2 ≤5	Lower epidermal growth factor level (plasma)	+
	Gonzalez-Redondo, 2012 [134]	Spain	85 PD	2.5	34.1	70.2	DSM IV	Increased periventricular hyperintensities (MRI)	+
	Compta, 2013 [122]	Spain	27 PD	1.5	30.0	69.0	MDS PDD	Low CSF amyloid β, impaired verbal learning, semantic fluency, visuoperception, frontal and anterior cingulate cortical thinning (MRI)	+
	Alves, 2014 [23]	Norway	104 PD	3.6	31.7	66.6	MDS PDD	Older age, MCI, low CSF amyloid β42 (ECL <376 pg/mL; ELISA <443 pg/mL)	+
								CSF amyloid β40, amyloid β38, total-tau, phosphorylated-tau	/
	Dubbelink, 2014 [116]	Netherlands	63 PD	7.0	38.1	62.4	MDS PDD	Combination of impaired fronto-executive task (spatial span) and low beta power (magnetoencephalography)	+
	Kandiah, 2014 [136]	Singapore	97 PD	1.5	29.9	65.3	MDS PDD	Hippocampal atrophy (MRI)	+
	Backstrom, 2015 [147]	Sweden	99 PD	5 to 9	41.4	71.3	MDS PDD	Higher CSF neurofilament light chain, heart fatty acid-binding protein, lower amyloid β1-42	+
	Latreille, 2016 [139]	Canada	68 PD, 44 control	4.01	32.4	64.9	MDS PDD	REM sleep and wakefulness slowing ratios in temporal and occipital areas, dominant occipital frequency (EEG)	+
	Lee, 2016 [131]	South Korea	96 PD	6.24	71.9	70.6	DSM IV	Postural instability	+

	Choi, 2017 [24]	South Korea	93 PD	6.7	52.7	65.9	MDS PDD	Older age, low metaiodobenzylguanidine (MIBG) uptake (H/M ratio < 1.35; ^{123}Iodine-MIBG myocardial scintigraphy)	+
	Modreanu, 2017 [133]	Spain	37 PD	1.5	38	64.0	MDS PDD	Non-motor predominance (≥3 non-motor symptoms with ≥2 being moderate-severe and ≥1 being present from onset), lower CSF amyloid β	+
	Delgado-Alvarado, 2018 [152]	Spain, US	320 PD, 240 control	3	35.6	62.1	MoCA<21/ MDS PDD	Higher CSF total-tau/α-synuclein, total-tau/amyloid ß1-42+α-synuclein	+
	Pilotto, 2018 [141]	Italy	54 PD	4	35.2	65.3	MDS PDD	Atypical FDG PET patterns, DLB/AD patterns (mainly characterized by metabolic alterations involving the posterior parietal-occipital regions; FDG PET)	+
	Lohle, 2019 [143]	Germany	29 PD	10.7	31.0	60.8	MoCA≤21	Striatal dopamine turnover (^{18}F-Fluorodopa PET)	/
	Massa, 2019 [124]	Italy	24 PD, 18 control	5.0	29.2	74.8	Not reported	Impaired Corsi span, semantic fluency, temporal lobe hypometabolism (FDG PET), reduced putamen/caudate (DAT SPECT)	+
	Chung, 2020 [142]	South Korea	205 PD	6.8	48.3	63.8	MDS PDD	More selective dopamine depletion in anterior putamen (^{18}F-FP-CIT PET)	+
	McCarter, 2020 [154]	US	25 PD	10	24	74.0	MDS PDD	Lower B12 (<587 ng/L; blood)	+
	Pereira, 2020 [137]	Sweden	106 PD, 42 control	5.5	35.9	61.3	MDS PDD, DSM V	Basal forebrain Ch4 atrophy (MRI)	+
								Ch1/Ch2, hippocampal, whole brain gray matter volume (MRI)	/
	Ma, 2021 [153]	US	301 PD, 144 control	6.4	35.9	62.1	MoCA<22	Higher serum neurofilament light chain	+
	Nicoletti, 2021 [108]	Italy	139 PD	2.0	37.3	65.7	MDS PDD	Male sex, MCI, higher Wahlund score for presence of white matter lesions (MRI)	+
	Backstrom, 2022 [81]	Sweden	143 PD	10	40.6	71.2	MDS PDD	Model including MCI, worse motor impairment, hyposmia, lower CSF amyloid β42	+

	Chung, 2022 [127]	South Korea	163 PD	4.2	54	70.4	MDS PDD	Higher motor reserve based on higher UPDRS-III and dopamine transporter availability in the posterior putamen (^{18}F-FP-CIT PET)	-
	Jeong, 2022 [41]	South Korea	571 PD	6.0	51.1	64.0	MDS PDD	Male sex, increased total white matter hyperintensities (MRI), reduced dopamine transporter availability in anterior putamen (^{18}F-FP-CIT PET)	+
	Myers, 2022 [55]	US	152 PD, 52 control	4.1	38.8	66.7	Clinical Dementia Rating≥1	CSF, PET amyloid β positivity (^{11}C-Pittsburgh Compound-B PET), *APOEe4*	+
	Sheng, 2022 [149]	US	174 PD, 85 control	6.7	33.3	60.6	MoCA<22	Higher CSF neurofilament light chain	+
	Vijiaratnam, 2022 [53]	UK	258 PD	3.7	36.3	68.4	MDS PDD	Older age, male sex, *APOEe4*, motor impairment, higher serum neurofilament light chain	+
								GBA	/
	Chung, 2023 [144]	South Korea	226 PD	4.9	48.2	70.6	MDS PDD	Low cingulate island sign ratio (posterior cingulate/ precuneus and cuneus uptake <0.9998 or 1 standard deviation below the mean of controls; ^{18}F-FP-CIT PET)	+
	Imarisio, 2023 [140]	Italy	49 PD	8	36.4	65.2	MDS PDD	Atypical FDG PET (AD-like, DLB-like) pattern vs typical PD-like pattern	+
	Jeong, 2023 [135]	South Korea	240 PD, 80 control	7.4	50.4	67.9	MDS PDD	Increased choroid plexus volume (MRI); relationship with dementia mediated by frontal/executive function	+
	Jeong, 2023 [145]	South Korea	394 PD	4.0	51.6	70.5	MDS PDD	Higher cerebral perfusion (^{18}F-FP-CIT PET)	+
	Lee, 2023 [155]	South Korea	124 PD	5.3	55.6	74.1	MDS PDD	Higher levodopa equivalent daily dose, higher UPDRS III, lower MMSE, lower serum zinc	+
	Liu, 2023 [150]	US	210 PD	6.4	35.2	60.9	MoCA<22	Higher CSF glial fibrillary acidic protein	+
	McFall, 2023 [34]	Canada	48 PD	3.0	43.8	71.5	DSM IV	Model with older age, worse gait, activities of daily living, Trail Making Test A, B, Choice reaction time, word recall, larger third ventricle volume (MRI), up-regulated metabolites	+

								(Hydroxy-isoleucine, His-Asn-Asp-Ser, Alanyl-alanine, Putrescine [-2H], 3,4-Dihydroxyphenylacetone) and creatinine (blood)	
	Oftedal, 2023 [151]	Norway	117 PD, 50 control	7.1	35.0	67.2	MDS PDD	Reduced CSF glucocerebrosidase activity	+
	Park, 2023 [32]	South Korea	262 PD	7.1	48.9	68.2	MDS PDD	Model with older age, disease duration, lower composite scores of visuospatial/visual memory, verbal memory, frontal/executive function, increased caudate texture heterogeneity (MRI)	+
	Chung, 2024 [146]	South Korea	397 PD	4.2	51.4	70.3	MDS PDD	Reduced uptake in frontal, parietal, temporal, lateral occipital regions vs relatively preserved cortical uptake (^{18}F-FP-CIT PET)	+
	Cousins, 2024 [148]	US	364 PD, 168 control	5	36.5	62.4	MoCA ≤20	Lower CSF amyloid β42, higher phosphorylated-tau181, higher serum neurofilament light chain	+
DLB	Dang-Vu, 2012 [171]	Canada	20 RBD, 10 control	3.1	20	67.9	Not reported	Increased hippocampal perfusion (^{99m}Tc-ethyl cysteinate dimer SPECT)	+
	Iranzo, 2014 [172]	Austria, Spain	55 RBD	5	14.5	68.9	McKeith 2005	Hyperechogenicity of the substantia nigra (transcranial sonography)	+
	Liu, 2019 [173]	Hong Kong	216 RBD	5	22.7	67.5	McKeith 2005	Severe phasic electromyogram (EMG) activity vs mild	+
	Laguna, 2021 [176]	Spain	33 RBD, 29 control	4.7	15.2	74.2	McKeith 2017	Serum area glycB (N-acetylneuraminic acid in glycoproteins)	+
	Rahayel, 2021 [175]	Australia, Canada	76 RBD	3.4	17	66.7	McKeith 2017, DSM V	Model with clinical score (MCI, akinetic-rigid motor phenotype) and brain deformation signature (atrophy in the basal ganglia, thalamus, amygdala, frontotemporal grey and white matter, subarachnoid/ventricular expansion; MRI)	+
	Miyamoto, 2022 [174]	Japan	87 RBD	3.5	36.4	70.8	McKeith 2017	Lower striatal binding ratio side (DAT SPECT)	/

	Fernandes, 2024 [177]	Italy	34 RBD, 33 control	7.6	17.6	67.1	McKeith 2017	Lower CSF amyloid β42 (<500 pg/mL)	+
								CSF phosphorylated-tau, total-tau, CSF/serum albumin	/
LBD	Numahata, 2021 [180]	Japan	30 RBD	6.4	23.3	70.2	McKeith 2017, DSM V	Reduced perfusion flow in precuneus, posterior cingulate, parietal association cortex (^{99m}Tc-ethyl cysteinate dimer SPECT)	+
								Cingulate island sign (^{99m}Tc-ethyl cysteinate dimer SPECT)	/

[a]Association with PDD, DLB, or LBD marked with - for reduced risk, + for increased risk, / for no significant association.
CSF: cerebrospinal fluid, DLB: dementia with Lewy bodies, DAT SPECT: dopamine transporter single photon emission computed tomography, DSM: The Diagnostic and Statistical Manual of Mental Disorders, EEG: electroencephalogram, FDG PET: fludeoxyglucose positron emission tomography, ICD: International Classification of Diseases, LBD: Lewy body dementia, MCI: mild cognitive impairment, MDS: International Parkinson and Movement Disorder Society, MMSE: Mini Mental State Exam, MoCA: Montreal Cognitive Assessment, PD: Parkinson's disease, PDD: Parkinson's disease dementia, PET: positron emission tomography, RBD: rapid eye movement sleep behavior disorder, SPECT: single-photon emission computed tomography, UPDRS: Unified Parkinson's Disease Rating Scale
